# Copper Single-Atoms Loaded on Molybdenum Disulphide Drive Bacterial Cuproptosis-Like Death and Interrupt Drug-Resistance Compensation Pathways

**DOI:** 10.1007/s40820-025-01955-2

**Published:** 2026-01-11

**Authors:** Wenqi Wang, Xiaolong Wei, Bolong Xu, Hengshuo Gui, Yan Yan, Huiyu Liu, Xianwen Wang

**Affiliations:** 1 College and Hospital of Stomatology, Anhui Medical University, Key Lab of Oral Diseases Research of Anhui Province, Hefei, 230032 People’s Republic of China; 2 School of Pharmaceutical Sciences, Anhui Medical University, Hefei, 230032 People’s Republic of China; 3 School of Biomedical Engineering, Anhui Medical University, Hefei, 230032 People’s Republic of China; 4 Beijing Advanced Innovation Center for Soft Matter Science and Engineering, State Key Laboratory of Organic‒Inorganic Composites, Bionanomaterials and Translational Engineering Laboratory, Beijing Key Laboratory of Bioprocess, Beijing Laboratory of Biomedical Materials, Beijing University of Chemical Technology, Beijing, 100029 People’s Republic of China; 5 School of Chemistry and Chemical Engineering, Anhui University of Technology, Ma’anshan, Anhui 243002 People’s Republic of China

**Keywords:** Nanozyme, Cuproptosis-like death, Bacterial resistance, Compensatory pathway, Wound healing

## Abstract

The development of highly efficient and multifunctional nanozymes holds promise for addressing the challenges posed by drug-resistant bacteria. Here, copper single-atom-loaded MoS_2_ nanozymes (Cu SAs/MoS_2_) were developed to effectively combat drug-resistant bacteria by synergistically integrating the triple strategies of oxidative damage, cuproptosis-like death and disruption of cell wall synthesis. Density functional theory revealed that each Cu center coordinated with three sulfur ligands, enhancing the adsorption of H_2_O_2_, which reduced the activation energy of the key step by 17%, thereby improving peroxidase-like (POD-like) activity. The generation of reactive oxygen species in combination with Cu SAs/MoS_2_ glutathione peroxidase-like (GSH-Px-like) for glutathione scavenging resulted in an imbalance in redox homeostasis within bacteria. Cu SAs/MoS_2_, which act as nanopioneers, drive oxidative stress to initiate the process of cuproptosis-like death, leading to abnormal aggregation of lipoylated proteins and inactivation of iron‒sulfur cluster proteins. Moreover, Cu SAs/MoS_2_ inhibited the biosynthesis of the peptidoglycan synthesis precursors d-glutamate and m-diaminopimelic acid and disrupted the peptidoglycan cross-linking process mediated by penicillin-binding proteins, effectively blocking the compensatory cell wall remodeling pathway of β-lactam-resistant bacteria. Overall, Cu SAs/MoS_2_ with multiple functions can not only efficiently kill bacteria but also decelerate the development of bacterial resistance to combat drug-resistant bacterial infections.

**Supplementary Information:**

The online version contains supplementary material available at 10.1007/s40820-025-01955-2.

## Highlights

- Peroxidase-like and glutathione peroxidase-like activities were significantly enhanced by atomic-level doping of Cu SAs/MoS_2_, which efficiently generated reactive oxygen species (ROS) and caused oxidative damage to drug-resistant bacteria.
- The ROS storms generated by single-atom-loaded MoS_2_ nanozymes (Cu SAs/MoS_2_) altered bacterial membrane permeability and facilitated Cu^2+^ entry into bacteria, enhancing bacterial cuproptosis-like death.
- Cu SAs/MoS_2_ interferes with bacterial energy metabolism and cell wall synthesis and inhibits peptidoglycan synthesis, weakening bacterial adaptation and drug resistance.

**Supplementary Information:**

The online version contains supplementary material available at 10.1007/s40820-025-01955-2.

## Graphical abstract

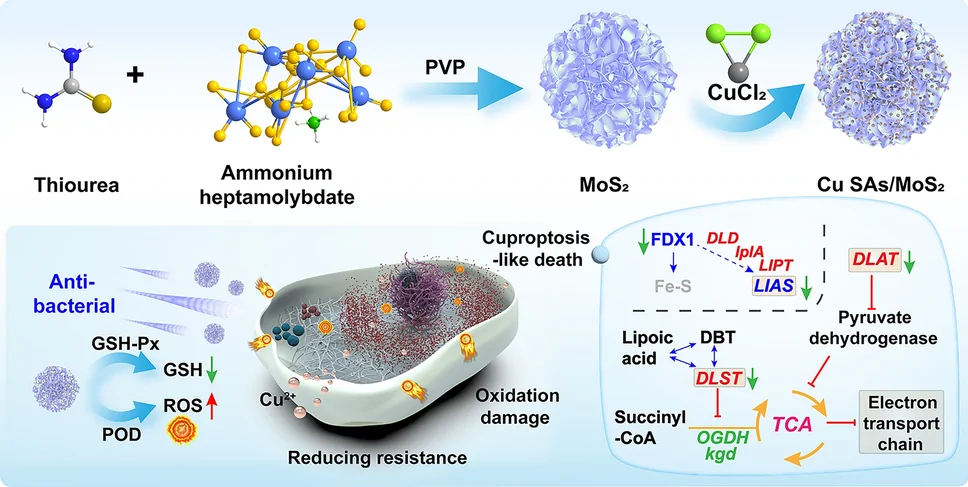

## Introduction

Bacterial antibiotic resistance represents a significant challenge to global public health [1–3]. As bacteria develop resistance to specific antibiotics, they evolve complex resistance mechanisms, making infections increasingly difficult to treat [4–7]. For example, methicillin-resistant *Staphylococcus aureus* (*MRSA*) evades the action of β-lactam antibiotics by altering the structure of penicillin-binding proteins (PBPs), reducing their affinity for these drugs, or producing β-lactamases that directly degrade antibiotics [8–10]. Notably, bacteria can acquire compensatory mutations that enable them to survive and proliferate in the presence of antibiotics [11–14]. F. Coll et al. reported that compensatory mutations lead to extensive protein variations in metabolic genes, quorum-sensing regulatory genes (e.g., *agrC* and *agrA*), and known antibiotic targets (e.g., *pbp2*, *dfrA*, *ileS*, and *fusA*) [15]. These findings suggest that inhibiting these compensatory metabolic pathways could weaken the effects of compensatory mutations and reduce bacterial resistance. Therefore, interventions targeting the mechanisms of compensatory mutations may serve as effective strategies to curb the development of antibiotic resistance. Notably, the multifunctionality and designability of nanozymes enable them to simultaneously target multiple bacterial pathways, significantly reducing the likelihood of bacteria developing resistance through compensatory mutations [16–18]. Additionally, the physicochemical stability of nanozymes far exceeds that of natural enzymes, allowing them to maintain their catalytic activity in complex environments (e.g., within biological systems), thereby increasing their practical applicability [19–21].

Nanozymes are a class of nanomaterials that mimic the catalytic activity of natural enzymes, and their core advantage lies in their ability to catalyze specific chemical reactions, particularly demonstrating significant antibacterial potential through oxidative damage [22–24]. Nanozymes can rely on POD activity, enabling them to catalyze the decomposition of H_2_O_2_ to produce highly reactive ·OH, which triggers oxidative stress and leads to bacterial death [25–27]. Among these materials, copper single-atom (Cu SAs) and molybdenum disulfide (MoS_2_) have shown exceptional efficacy in antibacterial therapy based on chemodynamic therapy (CDT) [28–31]. Owing to its unique atomic-level dispersion structure, Cu SAs exhibits extremely high catalytic activity and selectivity, significantly enhancing the efficiency of CDT [32, 33]. Moreover, as a two-dimensional nanomaterial with excellent biocompatibility and a large specific surface area, MoS_2_ can effectively load and stabilize Cu SAs to further enhance its catalytic performance [34, 35]. Additionally, copper-based nanozymes can induce the oligomerization of lipoylated proteins such as dihydrolipoamide acetyltransferase (DLAT), dihydrolipoamide succinyltransferase (DLST), and lipoic acid synthetase (LIAS) through the release of copper ions, leading to cellular metabolic dysfunction and ultimately triggering cuproptosis-like bacterial death [36–38]. However, cuproptosis-like mechanisms face challenges such as copper ion toxicity, difficulties in distribution regulation, and the potential for adaptive resistance in bacteria [39, 40]. Furthermore, while CDT can generate reactive oxygen species (ROS) to induce bacterial oxidative damage, its efficacy is limited by bacterial antioxidant defense mechanisms, and high concentrations of ROS may cause toxicity to host cells while potentially inducing bacterial resistance [41–43]. Therefore, a multimechanism therapeutic strategy combining CDT (oxidative damage) and cuproptosis-like treatment is expected to overcome the shortcomings of monotherapy, improve antibacterial efficacy, reduce the likelihood of bacterial resistance development, and optimize the safety of the treatment. Nevertheless, the dependence of antibacterial activity on copper ions, potential toxicity, and efficient ROS generation, as well as the role of oxidative damage in cuproptosis-like bacterial death, the extent of oxidative damage, and the underlying molecular mechanisms, need to be further explored. In short, this multipathway antibacterial mode of action is expected not only to make it more difficult for bacteria to develop resistance through compensatory mutations but also to enable them to exhibit greater broad-spectrum and selectivity in the face of resistant strains.

**Scheme 1:**
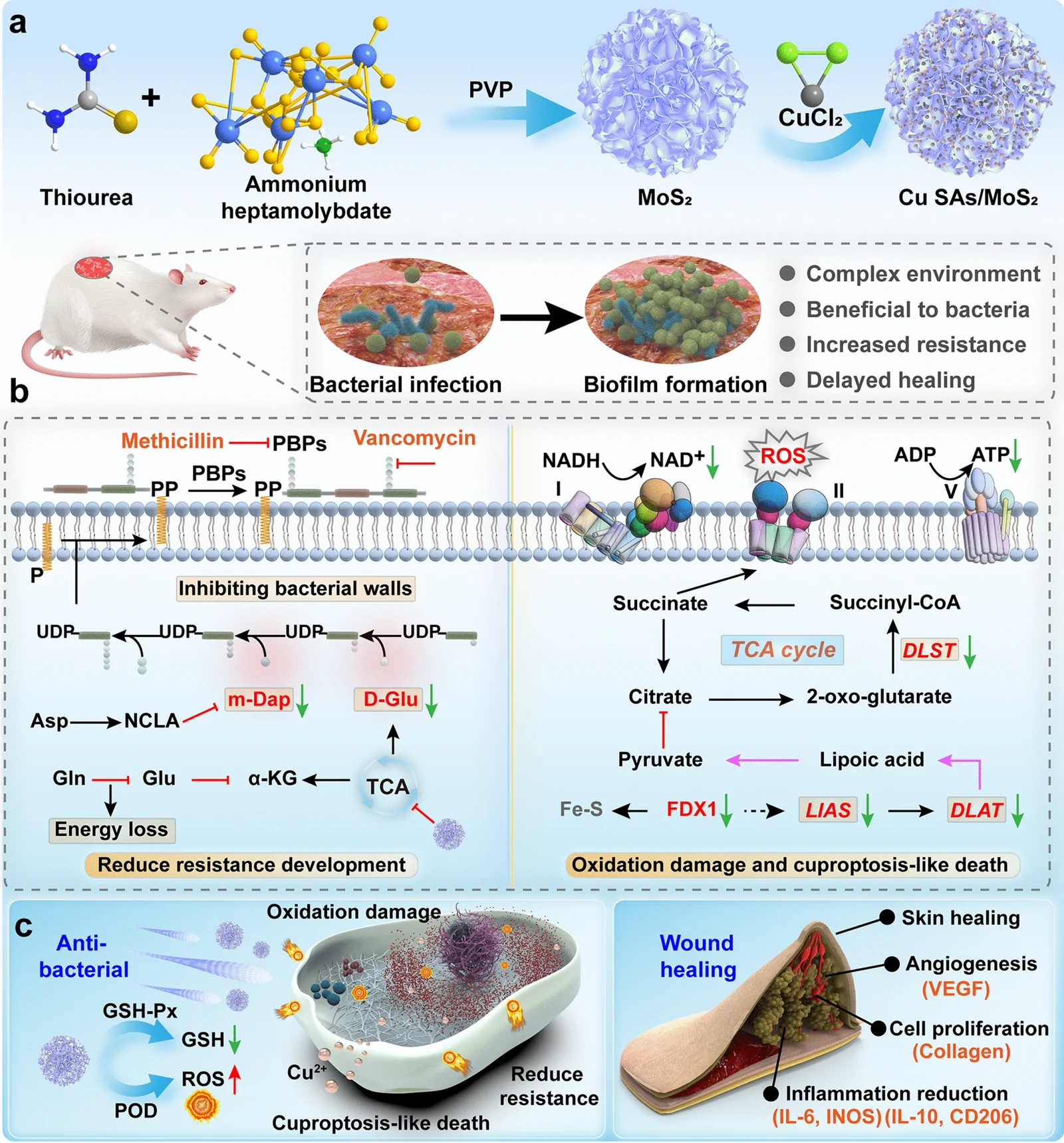
Cu SAs/MoS_2_ for infected wound treatment. **a** Synthesis process of Cu SAs/MoS_2_ and the wound infection process. **b** Inhibition of bacterial resistance development, oxidative damage, and cuproptosis-like death mechanisms by Cu SAs/MoS_2_. **c** In vivo antibacterial activity and promotion of wound healing

Here, a multifunctional nanozyme (Cu SAs/MoS_2_) was developed via a solution impregnation method for the treatment of drug-resistant bacterium-infected wounds by increasing oxidative damage and cuproptosis-like death (Scheme 1). Specifically, Cu SAs provide abundant active sites and an optimized electronic structure, which are introduced atomically into MoS_2_, thereby increasing the catalytic efficiency and synergistically increasing the POD-like and GSH-Px-like activities of Cu SAs/MoS_2_. Density functional theory (DFT) calculations revealed that Cu doping in the catalytic cycle enhances H_2_O_2_ adsorption and reduces the activation energy of the rate-determining step, confirming that Cu synergizes with S to provide superior POD-like activity for Cu SAs/MoS_2_. Notably, Cu SAs/MoS_2_ generates a significant amount of ROS through enhanced POD-like activity, causing oxidative damage to bacteria. Simultaneously, ROS alter bacterial membrane permeability, increasing the influx of Cu^2^⁺ into bacteria and inducing cuproptosis-like bacterial death. In this process, Cu^2^⁺ binds to lipoylated proteins (DLAT, DLST, LIAS) in the TCA cycle, triggering their abnormal oligomerization and reducing the levels of iron‒sulfur cluster proteins, thereby inducing proteotoxic stress and leading to bacterial cuproptosis-like death. Furthermore, Cu SAs/MoS_2_ inhibits energy metabolism and cell wall synthesis, interfering with compensatory mechanisms of bacterial resistance. This leads to a reduction in the supply of materials and energy required for cell wall synthesis, while the suppression of genes such as *GudB* and *GDH2* decreases the levels of key peptidoglycan cross-linking components (D-glutamate and m-diaminopimelic acid), which inhibits peptidoglycan synthesis and diminishes bacterial adaptability and resistance.

## Experimental Section

### Materials

Copper chloride(II) (CuCl_2_; 98%), ammonium heptamolybdate ((NH_4_)_6_Mo_7_O_24_; 99.99%), thiourea (NH_2_CSNH_2_; 99.98%), and polyvinyl pyrrolidone (PVP; K30) were purchased from Sigma‒Aldrich. Ethanol (99.9%) was obtained from Shanghai Chemical Reagent Company. All chemicals were used as received without additional purification. Ar was sourced from Nanjing Special Gas Company. All aqueous solutions were prepared using deionized water with a resistivity of 18.2 MΩ cm^−1^.

### Synthesis of MoS_2_

In our synthetic procedure, 617 mg (0.5 mmol) of ammonium molybdate ((NH_4_)_6_Mo_7_O_24_), 533 mg (7 mmol) of thiourea (NH_2_CSNH_2_), and 300 mg (0.005 mmol) of PVP were dissolved in 17 mL of deionized water under vigorous stirring to form a homogeneous solution. The resulting solution was then transferred into a 50 mL Teflon-lined stainless-steel autoclave and maintained at 220 °C for 18 h. After cooling to room temperature, the generated precipitates were collected by centrifugation and washed thoroughly with water and ethanol several times. The final products were dried under vacuum at 50 °C for 12 h.

### Synthesis of Cu SAs/MoS_2_

For the synthesis of Cu SAs/MoS_2_, 160 mg of MoS_2_ was dispersed in 160 mL of a water‒ethanol mixture (with a volume ratio of 9:1) at 90 °C. Subsequently, 20 mL of a 0.5 mmol L^−1^ CuCl_2_ solution was gradually added into the beaker via a peristaltic pump at a rate of 20 min mL^−1^. After the injection, the solution was allowed to cool to room temperature, followed by filtration. The resulting product was dried under vacuum at 50 °C for 12 h and then annealed in an argon atmosphere at 300 °C for 3 h.

### In vitro Nanozyme Activity

Cu SAs/MoS_2_ (150 μg mL^−1^) was added to a 24-well plate. H_2_O_2_ solutions were subsequently added to each well at concentrations of 0, 0.02, 0.04, 0.06, 0.08, and 0.1 mM. Then, 100 mM TMB or 100 mM OPD substrate solution was added to each well. The well plate was placed at room temperature (25 °C) and allowed to react in the dark for 15 min. After the reaction was complete, the absorbance was measured via a spectrophotometer at wavelengths of 450 nm (TMB) and 492 nm (OPD). In a 0.1 mM H_2_O_2_ solution, Cu SAs/MoS_2_ solutions with concentrations of 0, 25, 50, 75, 100, and 150 μg mL^−1^ were added. Then, the TMB or OPD substrate mixture was added, and the reaction was carried out at room temperature in the dark for 15 min. After the reaction was complete, the absorbance was measured via a spectrophotometer at wavelengths of 450 and 492 nm.

The experiments included a positive control group, a negative control group, a PBS group, an H_2_O_2_ group, a MoS_2_ + H_2_O_2_ group, and a Cu SAs/MoS_2_ + H_2_O_2_ group. The concentration of H_2_O_2_ was 0.1 mM, and the concentrations of both the Cu SAs/MoS_2_ and MoS_2_ were 150 μg mL^−1^. Each sample was added to a 10-mM GSH solution in turn, and then 10 μL of a 1 mM DTNB solution was added to each well. After sample addition, the well plate was incubated in a 37 °C constant-temperature water bath for 10 min. After incubation, the absorbance was measured via a spectrophotometer at a wavelength of 412 nm. Cu SAs/MoS_2_ at concentrations of 50 and 150 μg mL^−1^ was used to carry out the above steps, and the absorbance at a wavelength of 412 nm was detected over a time gradient.

### DFT Calculations

All periodic DFT computations were performed via the Vienna Ab initio Simulation Package (VASP), which employs a rigorously tested computational protocol [44]. The electron exchange‒correlation interactions were modeled with the Perdew–Burke–Ernzerhof (PBE) generalized gradient approximation (GGA) functional, whereas the core‒electron interactions were treated via the projector augmented wave (PAW) method with explicit consideration of the valence electron configurations [45, 46]. To ensure numerical precision, we implemented the following convergence criteria: (1) A plane-wave basis set cutoff energy of 450 eV was selected on the basis of systematic convergence tests spanning 400–500 eV; (2) electronic self-consistency was achieved with a tight threshold of 1 × 10^–5^ eV per atom; and (3) ionic relaxation proceeded until residual atomic forces fell below 0.001 eV Å^−1^, ensuring optimized geometric configurations. This combination of parameters guarantees energy fluctuations below 0.01 eV atom^−1^ in our test systems.

### In Vitro Antibacterial Evaluation

*MRSA* (Mu50) and *E. coli* (DH5α) were cultured in Luria–Bertani (LB) media. The control group, H_2_O_2_ group, MoS_2_ + H_2_O_2_ group, and Cu SAs/MoS_2_ + H_2_O_2_ group were established. The bacterial concentration was 1 × 10^6^ CFU mL^−1^, the concentration of H_2_O_2_ was 0.1 mM, and the concentrations of MoS_2_ and Cu SAs/MoS_2_ were 150 μg mL^−1^. After the corresponding substances were added according to the grouping, the samples were cultured in a shaker at 37 °C and 180 r min^−1^ for 12 h. The absorbance values were subsequently measured via a spectrophotometer at a wavelength of 600 nm every hour. After 12 h of treatment, the bacterial solutions were cultured via the plate-spreading method and incubated in an oven at 37 °C for 15 h. Then, photographs were taken, and statistical analysis was performed via ImageJ. Furthermore, the bacteria were treated with Cu SAs/MoS_2_ at concentrations of 0, 25, 50, 75, 100, and 150 μg mL^−1^ according to the abovementioned steps, and photographs were taken for recording.

### ROS Detection

The bacteria were treated according to the control group, H_2_O_2_ group, MoS_2_ + H_2_O_2_ group, or Cu SAs/MoS_2_ + H_2_O_2_ group, following the experimental steps in the in vitro antibacterial evaluation. DCFH-DA (Beyotime) was diluted at a ratio of 1:1000, 10 μL of the diluted solution was added to each treatment mixture, and the mixture was incubated in the dark for 20 min. A laser scanning confocal microscopy imaging system (CSIM-130, Beijing Century Sunny Technology Co., Ltd.) was subsequently used for photographic recording.

### Prokaryotic Transcriptome Sequencing and Metabolomics Analysis

*MRSA* was treated with Cu SAs/MoS_2_ (150 μg mL^−1^) + H_2_O_2_ (0.1 mM) for 4 h, with three replicates. The testing was carried out by Shanghai Personalbio Technology Co., Ltd. (China), and the sample measurement data were analyzed by Shanghai Keyijing Biotechnology Co., Ltd. (China).

### Copper Ion Detection

Inductively coupled plasma‒mass spectrometry (ICP‒MS) was used to detect copper ions in the Cu SAs/MoS_2_. The copper standard solution was serially diluted with a 2% nitric acid solution to prepare standard working solutions at different concentrations, such as 0, 1, 5, 10, 50, and 100 μg L^−1^. These standard working solutions were introduced into the instrument to measure the signal intensity, and a standard curve was drawn. The prepared Cu SAs/MoS_2_ solution was then introduced into the instrument for measurement, and the copper ion content in the sample was calculated on the basis of the standard curve.

### Detection of Bacterial Resistance Development

Minimum inhibitory concentration (MIC) assay: Three to five morphologically consistent single colonies of *MRSA* were selected and suspended in 2–3 mL of sterile physiological saline. The mixture was then vortexed to ensure uniformity, and the turbidity was adjusted to 0.5 McFarland (approximately 1 × 10^8^ CFU mL^−1^). The suspension was then diluted 1:100 with broth to achieve a final bacterial concentration of approximately 5 × 10^5^ CFU mL^−1^. A concentration gradient of 2.5, 5, 10, 15, 20, and 25 μg mL^−1^ of Cu SAs/MoS_2_ was established for the bacterial suspension. The samples were incubated at 37 °C for 20 h. Visible bacterial growth was assessed visually, and the absorbance at 600 nm was recorded via a spectrophotometer. The MIC was determined as the lowest concentration that completely inhibited visible bacterial growth.

The assessment of resistance development was performed via a ‘drug concentration escalation‒continuous passage’ strategy. Specifically, the parent strain (*MRSA*) was used as the starting point, and its minimum inhibitory concentration (MIC_0_) was determined via the MIC detection method described above, which served as the baseline for subsequent comparisons. The strain was subsequently inoculated into broth containing 0.5 × MIC_0_ Cu SAs/MoS_2_ (8.5 μg mL^−1^) and cultured for 24 h. The surviving bacterial suspension was then retested for MIC. This process is repeated every 24 h, and the MIC is recorded over a 21-day period. The same method was used to evaluate the development of resistance in MoS_2_, vancomycin, and methicillin.

The long-term effects of the intermittent use of Cu SAs/MoS_2_ on bacterial resistance patterns are similar to the steps described above. After the MIC_0_ of the parental strain was determined, the strain was inoculated into broth containing 0.5 × MIC_0_ Cu SAs/MoS_2_ (8.5 μg mL^−1^) and cultured for 24 h. The surviving bacterial suspension was then retested to determine the MIC. Two rounds of drug-free passages (every 24 h) were subsequently performed. The passages were alternated between ‘drug‒drug-free‒drug-free’, and the MIC was recorded over 21 days to assess the development of resistance under intermittent dosing. The same method was used to assess the development of resistance under intermittent dosing for MoS_2_, vancomycin, and methicillin.

### Membrane Depolarization Analysis

DISC_3_(5) dye was used to detect bacterial membrane depolarization activity. The four resistant bacteria were removed and washed three times with PBS. A total of 900 μL of the bacterial suspension were mixed with 100 μL of DISC_3_(5) dye (diluted at a ratio of 1:1000) and incubated in the dark for 20 min. A laser scanning confocal microscopy imaging system was used for photographic recording.

### In Vitro Antibiofilm Ability

One hundred microlitres of bacteria (*MRSA* and *E. coli*) and 900 μL of LB medium were added to a 24-well plate and cultured in an oven at 37 °C for 72 h. The biofilms were subsequently treated with PBS, H_2_O_2_, MoS_2_ + H_2_O_2_, or Cu SAs/MoS_2_ + H_2_O_2_ for 4 h to disrupt them. Additionally, PBS, H_2_O_2_, MoS_2_ + H_2_O_2_, or Cu SAs/MoS_2_ + H_2_O_2_ was added to the bacterial cultures, which was subsequently incubated in an oven at 37 °C for 72 h to inhibit biofilm growth. Next, the treated biofilms were gently washed with PBS, and 500 μL of methanol was added for fixation for 30 min. After the methanol was removed, the biofilms were stained with 0.1% (w/v) crystal violet for 20 min. The biofilms in the wells were then photographed. Finally, 300 μL of 30% glacial acetic acid was added to dissolve the stained biofilms, and the absorbance at 595 nm was measured via a microplate reader.

### Cytotoxicity and Hemolysis Tests

A cryopreserved human keratinocyte cell line (HaCaT), human umbilical vein endothelial cells (HUVECs), a mouse macrophage line (RAW 264.7), and a mouse fibroblast line (L929) were cultured to the logarithmic growth phase. The cell concentration was adjusted, and the cells were seeded into a 96-well plate. After the cells had allowed to adhere to the plate for 24 h, they were treated with different concentrations (0, 50, 100, 200, 300, 400, and 500 μg mL^−1^) of Cu SAs/MoS_2_ and cultured for 24 h. Then, CCK-8 solution was added, and the plate was incubated. The absorbance was measured at 450 nm after incubation with CCK-8 solution via a microplate reader.

Eight-week-old BALB/c mice were selected, and their blood was collected. Five hundred microliters of red blood cells were resuspended in 450 μL of normal saline. Then, Cu SAs/MoS_2_ were added at concentrations of 0, 25, 50, 75, 100, and 150 μg mL^−1^, and the mixtures were incubated at 37 °C for 2 h. After incubation, the absorbance of the solutions was measured at 540 nm via a microplate reader.

### Assessment of Cu SAs/MoS_2_ Enzyme Activity in Purulent Inflammation

In vitro simulation of the inflammatory microenvironment: A total of 5 × 10^5^ macrophages (RAW264.7) were seeded into a 6-well plate and incubated at 37 °C for 6 h to allow them to adhere. The serum-free culture medium was then replaced, and the cells were stimulated with LPS (100 ng mL^−1^) combined with IFN-γ (20 ng mL^−1^) for 24 h to establish an inflammatory microenvironment. The supernatant was collected and stored for later use.

Subcutaneous abscess mouse model: Eight-week-old male BALB/c mice were randomly divided into four groups, shaved on the back, and disinfected with iodine. *MRSA* was suspended at a concentration of 1 × 10^8^ CFU mL^−1^ in 50 μL of PBS and mixed with an equal volume of sterile paraffin oil to prepare a bacterial suspension. Using a 1-mL insulin syringe, 100 μL of the mixture was slowly injected subcutaneously into the back, and the puncture site was gently pressed to close. Postoperative observations were conducted daily to monitor redness, swelling, and purulent exudate. A distinct abscess formed locally on day three, establishing the subcutaneous abscess model. On day seven after treatment, the skin from the abscess area was harvested for histological analysis.

### Wound Models and In Vivo Antibacterial Experiments

All animal experiments were reviewed and approved by the Animal Care and Use Committee of Anhui Medical University (No. LLSC20220731). A burn model of *MRSA* and *E. coli* infection was constructed to evaluate the antibacterial capacity and wound healing promotion capacity of Cu SAs/MoS_2_ in vivo. Male BALB/c mice at 8 weeks of age were anesthetized, and a 10-mm wound was created on the back using a burn device. The wound area was then promptly debrided. Fifty microliters of *MRSA* or *E. coli* (1 × 10^6^ CFU mL^−1^) were inoculated separately onto the wound surface and then cultured for 24 h. Treatment was carried out by instilling 150 μg mL^−1^ Cu SAs/MoS_2_. On the 6th day, the wounded skin was removed, placed in PBS, spread onto an agar plate and cultured for 15 h. On the 12th day, wound skin tissues were subsequently collected for eukaryotic transcriptome sequencing analysis, H&E and Masson staining, and fluorescence staining of multiple biomarkers. Moreover, mouse blood and organs (heart, liver, spleen, lungs, and kidneys) were collected for routine blood tests, blood biochemical tests, and H&E staining experiments to evaluate in vivo safety.

### Statistical Analysis

Comparisons of the means of the two groups were statistically analyzed via Student’s *t* test. To compare means between more than two groups, one-way analysis of variance (ANOVA) was used for normally distributed data. The data are expressed as the mean ± standard mean error (SEM); *P* < 0.05 was considered to indicate statistical significance (*P < 0.05, **P < 0.01, **P < 0.001).

## Results and Discussion

### Synthesis and Characterization of Cu SAs/MoS_2_

**Fig. 1:**
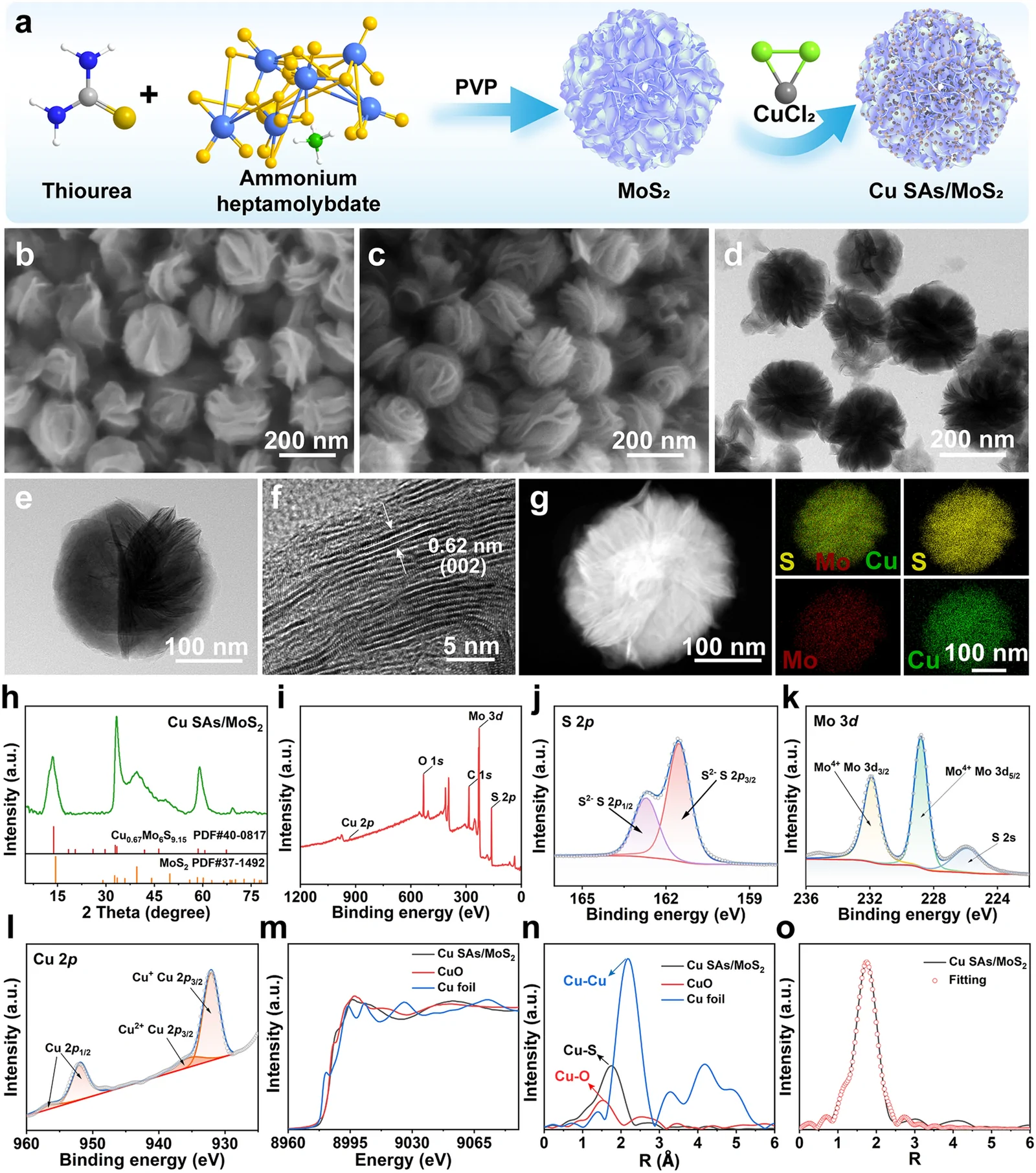
Characterization of Cu SAs/MoS_2_. **a** Schematic diagram of the principle of synthesis. SEM images of **b** MoS_2_ and **c** Cu SAs/MoS_2_. **d**,** e** TEM images. **f** HRTEM image of Cu SAs/MoS_2_. **g** HAADF-STEM image and elemental mapping images.** h** XRD pattern. **i** XPS survey spectrum of Cu SAs/MoS_2_. **j** S 2*p*, **k** Mo 3*d* and **l** Cu 2*p* XPS spectra of the Cu SAs/MoS_2._ **m** Cu K-edge XANES spectra and **n** FT-EXAFS spectra of the Cu foil, CuO, and Cu SAs/MoS_2_. **o** FT-EXAFS fitting curves

In this synthesis strategy, MoS_2_ nanoflowers were first prepared via a hydrothermal method, followed by the uniform loading of single Cu atoms onto MoS_2_ to form a Cu SAs/MoS_2_ nanozyme (Fig. 1a). Scanning electron microscopy (SEM) and transmission electron microscopy (TEM) revealed that MoS_2_ and Cu SAs/MoS_2_ have flower-like structures with uniform sizes, smooth surfaces, and no agglomeration (Fig. 1b–e). High-resolution TEM (HRTEM) clearly revealed lattice fringes of Cu SAs/MoS_2_ with a lattice spacing of 0.62 nm, corresponding to the (002) plane of MoS_2_ (Fig. 1f). Elemental mapping (EDX mapping) demonstrated the uniform distribution of Cu on the MoS_2_ nanoflowers, confirming the successful loading of the Cu SAs (Fig. 1g). In the X-ray diffraction (XRD) pattern, the characteristic diffraction peak of Cu SAs/MoS_2_ matched the standard spectrum of MoS_2_ (PDF#37-1492), with no peaks corresponding to metallic Cu compounds (PDF#40-0817), indicating that Cu was dispersed in a single-atom form (Fig. 1h). X-ray photoelectron spectroscopy (XPS) analysis further confirmed the chemical states of Cu SAs/MoS_2_ (Fig. 1i). In the Mo 3*d* spectrum, two characteristic peaks were observed at 229.7 and 232.9 eV, corresponding to Mo 3*d*_5/2_ and Mo 3*d*_3/2_, respectively, which are typical features of Mo(IV) (Fig. 1k). Additionally, the S 2*p* spectrum exhibited main peaks at 161.9 and 163.0 eV, corresponding to S 2*p*_3/2_ and S 2*p*_1/2_, respectively, indicating the presence of S(II) (Fig. 1j). The Cu 2*p* spectrum showed characteristic peaks at 932.5 eV (2*p*_3/2_) and 952.5 eV (2*p*_1/2_), indicating the presence of Cu(0) (Fig. 1l). To further analyze the fine atomic-scale structure of Cu SAs/MoS_2_, synchrotron-based X-ray absorption spectroscopy (XAFS) was performed. The Cu K-edge X-ray absorption near-edge structure (XANES) spectrum revealed that the absorption edge of Cu SAs/MoS_2_ lies between those of the Cu foil and CuO, suggesting a Cu valence state between 0 and + 2 (Fig. 1m). Furthermore, the Fourier transform extended X-ray absorption fine structure (FT-EXAFS) spectrum of Cu SAs/MoS_2_ exhibited a prominent Cu‒S coordination peak, with no Cu‒Cu or Cu‒O coordination peaks, indicating that the Cu atoms were primarily coordinated with the S atoms in MoS_2_, forming stable bonds and successfully dispersing as single atoms on the MoS_2_ surface (Fig. 1n). The fitting curve and parameters of FT-EXAFS revealed a Cu–S coordination number of 3 in Cu SAs/MoS_2_, confirming the formation of a Cu-S_3_ single-atom structure (Fig. 1o and Table S1). Thus, this synthesis strategy successfully produced MoS_2_-supported Cu SAs.

### Dual Enzyme-Like Activity of Cu SAs/MoS_2_

**Fig. 2:**
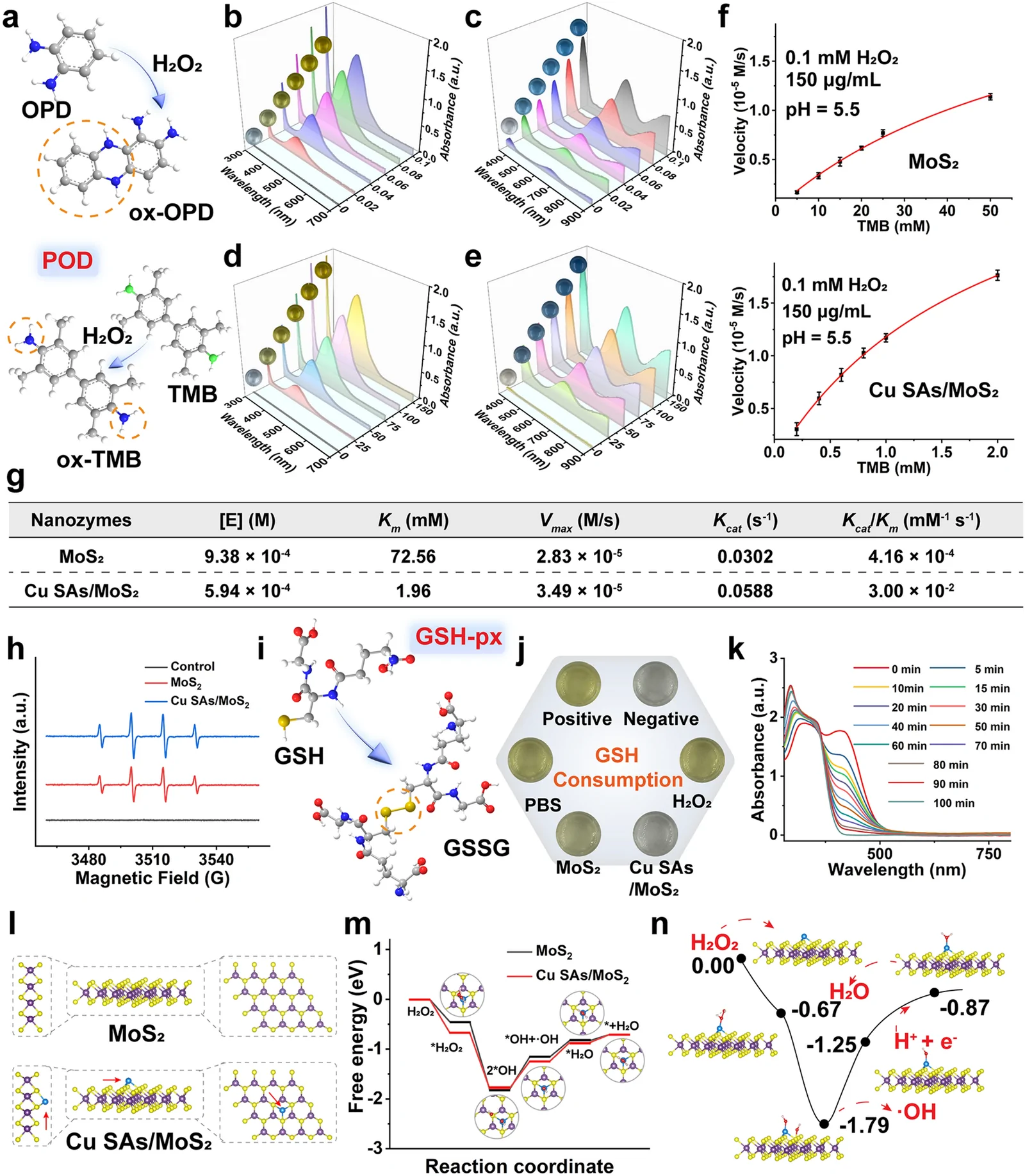
Enzyme-like activity of Cu SAs/MoS_2_ and DFT calculations. **a** Schematic diagram of the POD-like activity of Cu SAs/MoS_2_ using OPD and TMB. POD-like activity of Cu SAs/MoS_2_ at different H_2_O_2_ concentrations using **b** OPD and **c** TMB. POD-like activity at different Cu SAs/MoS_2_ concentrations using **d** OPD and **e** TMB. **f** Michaelis‒Menten kinetics of MoS_2_ and Cu SAs/MoS_2_.** g** Comparison of the kinetics of MoS_2_ and Cu SAs/MoS_2_. [*E*] is the molar concentration of the nanozymes. *K*_*m*_ is the Michaelis–Menten constant. *V*_max_ is the maximal reaction velocity. *K*_cat_ is the catalytic rate constant. The value of *K*_cat_/*K*_*m*_ represents the catalytic efficiency.** h** ESR spectra of ·OH. **i** Schematic diagram of the GSH-Px-like activity of Cu SAs/MoS_2_. **j** GSH consumption capacity of different groups. **k** Time-dependent GSH depletion of Cu SAs/MoS_2_ at a concentration of 50 μg mL^−1^ using the DTNB probe. **l** Atomic structure of MoS_2_ and Cu SAs/MoS_2_. **m** Free energy diagram for H_2_O_2_ decomposition catalyzed by MoS_2_ and Cu SAs/MoS_2_. **n** Schematic energy profile of Cu SAs/MoS_2_

Enzymatic functionality is a critical factor determining the therapeutic applications of nanozymes. Here, a series of experiments were designed to evaluate the enzyme-like activity and catalytic performance of Cu SAs/MoS_2_. First, the POD-like activity of Cu SAs/MoS_2_ was assessed by using two common enzyme substrates, 3,3′,5,5′-tetramethylbenzidine (TMB), and o-phenylenediamine (OPD) (Fig. 2a). Under the catalysis of Cu SAs/MoS_2_, the reaction with H_2_O_2_ produced yellow and blue products in the OPD and TMB working solutions, respectively, with characteristic peaks appearing at 492 and 650 nm, demonstrating typical POD-like activity. As the concentration of H_2_O_2_ increased, the color intensity of both products also increased, indicating the dependence of Cu SAs/MoS_2_ on the H_2_O_2_ concentration (Fig. 2b, c). Furthermore, the absorbance values gradually increased with increasing Cu SAs/MoS_2_ concentration (0–150 μg mL^−1^) (Fig. 2d, e). Although the absorbance changes were more pronounced for OPD, TMB also exhibited a similar trend, suggesting that Cu SAs/MoS_2_ possesses stable POD-like activity. The enzyme activity of MoS_2_ is slightly lower than that of Cu SAs/MoS_2_ (Fig. S1). To illustrate the effect of Cu SAs doping in MoS_2_ on POD-like activity, the Michaelis‒Menten kinetic model was used for quantitative analysis and comparison. The *V*ₘₐₓ of Cu SAs/MoS_2_ (3.49 × 10⁻^5^ M s^−1^) was significantly greater than that of MoS_2_ (2.83 × 10⁻^5^ M s^−1^), and the *Kₘ* of MoS_2_ (72.56 mM) was 37 times greater than that of Cu SAs/MoS_2_ (1.96 mM) (Fig. 2f). The increase in *V*ₘₐₓ and decrease in *Kₘ* indicate that Cu SAs/MoS_2_ has higher catalytic efficiency and stronger substrate affinity. In addition, ·OH was detected via the electron spin resonance (ESR) technique, and compared with MoS_2_, Cu SAs/MoS_2_ was found to be capable of generating ·OH more significantly (Fig. 2g). All the kinetic parameters of those nanozymes are presented in Fig. 2h. The kinetic parameters, including *V*ₘₐₓ, *K*_cat*,*_ and *K*_cat_/*Kₘ*, of Cu SAs/MoS_2_ were greater than those of MoS_2_. This phenomenon may be attributed to the doping of Cu SAs, which provide more adsorption sites for H_2_O_2_, thereby accelerating the electron transfer process and ultimately enhancing the overall catalytic efficiency.

Interestingly, 5,5′-dithiobis(2-nitrobenzoic acid) (DTNB) detection revealed that Cu SAs/MoS_2_ can scavenge GSH, resulting in GSH-Px-like activity (Figs. 2i, j and S2). At a Cu SAs/MoS_2_ concentration of 50 μg mL^−1^, GSH was completely depleted in the DTNB working solution within 100 min (Fig. 2k). Under the same conditions, when the concentration was increased to 150 μg mL^−1^, GSH was completely depleted within just 20 min (Fig. S3). Notably, in bacteria, high-GSH expression significantly enhances antioxidant defense capabilities by neutralizing ROS and reducing H_2_O_2_ concentrations, thereby mitigating oxidative stress. This enhanced antioxidant capacity may severely weaken the bactericidal effect of POD-like antibacterial agents, but the presence of GSH-Px-like activity can effectively address this issue. In short, Cu SAs/MoS_2_ not only releases a large amount of ROS through POD-like activity for antibacterial purposes but also scavenges GSH through GSH-Px-like activity, overcoming the resistance of high-GSH-expressing bacteria to oxidative antibacterial agents.

### DFT Calculations

To elucidate the distinct peroxidase-like catalytic mechanisms of MoS_2_ and Cu SAs/MoS_2_ at the atomic level, we performed systematic DFT calculations to characterize the reaction energetics at the Mo active sites in MoS_2_ and the Cu active sites in Cu SAs/MoS_2_. The MoS_2_ model was constructed with 4 × 4 × 1 primitive MoS_2_ cells with *a* = *b* = 12.66 Å and *γ* = 120°. On the basis of the complementary evidence from XANES analysis and XPS analyses, the Cu dopants in the surface-modified MoS_2_ exhibit a characteristic coordination number of three. This fundamental finding enables us to conclusively establish an atomic-scale structural model of Cu SAs/MoS_2_, wherein each Cu center adopts a trigonal planar geometry coordinated with three sulfur ligands (Fig. 2l).

The catalytic cycle begins with the adsorption and activation of H_2_O_2_ molecules (Fig. 2m, n). Our calculations reveal that pristine MoS_2_ has a Gibbs free energy of − 0.45 eV during H_2_O_2_ adsorption, whereas Cu-doped MoS_2_ has a significantly greater adsorption capability, with a more negative value of − 0.67 eV. This marked improvement in adsorption strength indicates that the incorporation of Cu effectively optimizes the electronic structure of the catalyst surface. Upon adsorption, the H_2_O_2_ intermediate spontaneously dissociates into two hydroxyl radicals (·OH), and the free energies of dissociation were calculated to be − 1.36 and − 1.12 eV for MoS_2_ and Cu SAs/MoS_2_, respectively. Notably, while both systems thermodynamically favor this dissociation step, the energy difference suggests distinct stabilization mechanisms between the two catalysts. The rate-determining step (RDS) involves the desorption of one ·OH radical into solution while retaining the other ·OH for subsequent reactions. Critical analysis of the energy barriers revealed that, compared with pristine MoS_2_, Cu SAs/MoS_2_ requires only 0.54 eV to overcome this RDS. This 17% reduction in activation energy directly correlates with the enhanced ·OH radical generation efficiency observed experimentally. The retained ·OH intermediate then undergoes protonation to form H_2_O, with associated energy barriers of 0.33 eV (MoS_2_) and 0.38 eV (Cu SAs/MoS_2_), respectively. Finally, H_2_O desorption completes the catalytic cycle, requiring 0.12 and 0.16 eV for MoS_2_ and Cu SAs/MoS_2_, respectively. Taken together, these computational insights corroborate the experimental observation of superior peroxidase-like activity in Cu SAs/MoS_2_, ultimately attributed to the synergistic interplay between the Cu and S atoms in modulating the reaction energetics.

### Antibacterial Activity of Cu SAs/MoS_2_ In Vitro

**Fig. 3:**
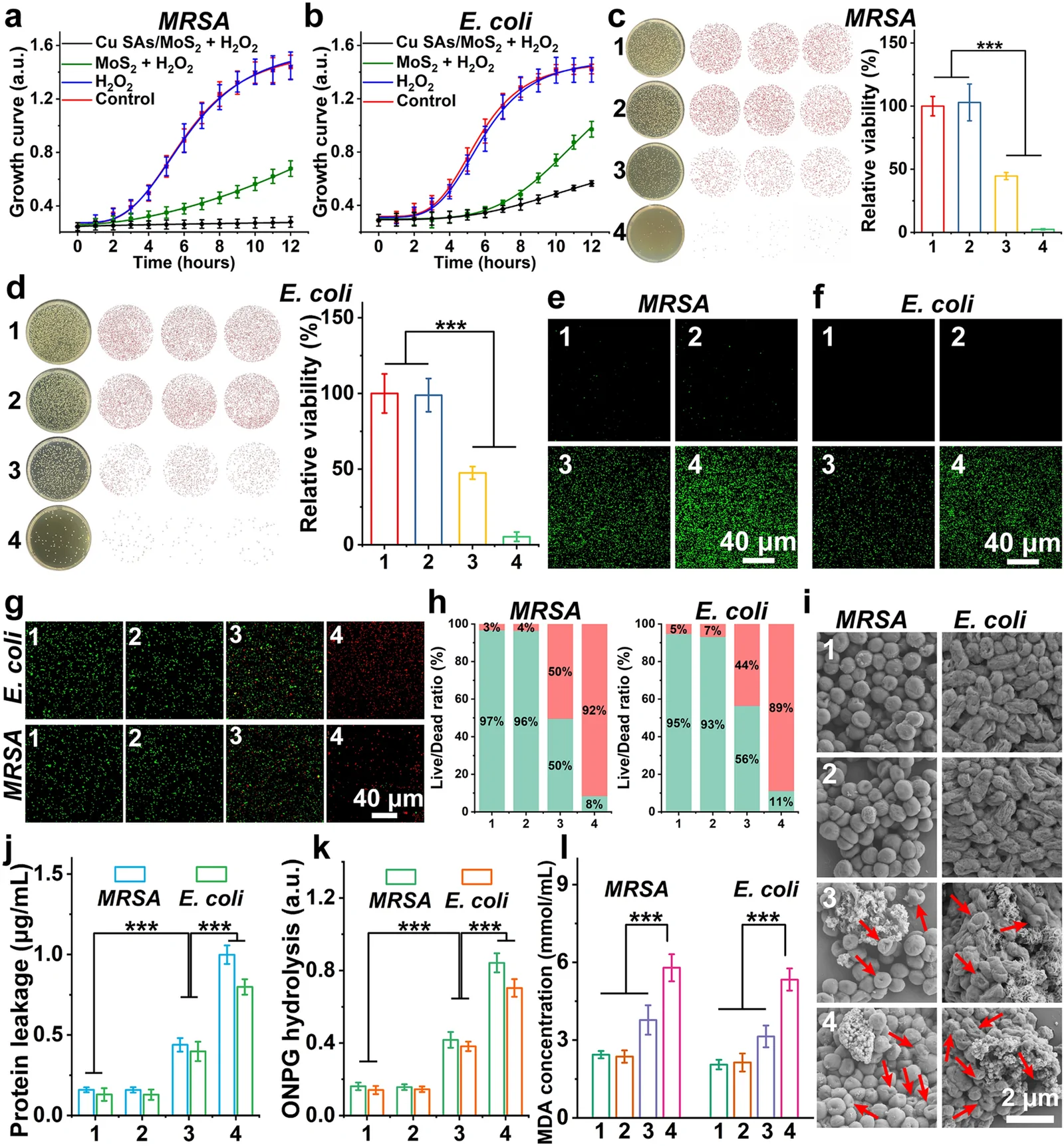
In vitro antibacterial performance of Cu SAs/MoS_2_. Growth curves of the four groups of **a** *MRSA* and **b** *E. coli*. Plate photographs and colony counts for **c** anti-*MRSA* and **d** anti-*E. coli* activity of Cu SAs/MoS_2_. CLSM fluorescence images of ROS in **e** *MRSA* and **f** *E. coli* subjected to different treatments via DCFH-DA. Fluorescence images **g** and live‒dead ratio analysis **h** of CLSM live‒dead cells after *MRSA* and *E. coli* treatment. **i** SEM images of bacteria after four treatments. **j** Protein leakage of bacteria after different treatments. **k** Permeability of the bacterial cell membrane. **l** MDA levels after different treatments. *Note*: 1: Control; 2: H_2_O_2_; 3: MoS_2_ + H_2_O_2_; 4: Cu SAs/MoS_2_ + H_2_O_2_

Next, given the high POD-like and GSH-Px-like activities caused by the doping of Cu SAs into the nanoflower structure of MoS_2_, the in vitro antibacterial performance of Cu SAs/MoS_2_ was evaluated. *MRSA* and *E. coli* were exposed to four treatment conditions for 12 h: Cu SAs/MoS_2_ (150 μg mL^−1^) + H_2_O_2_ (0.1 mM), MoS_2_ (150 μg mL^−1^) + H_2_O_2_ (0.1 mM), H_2_O_2_ (0.1 mM), and broth solution. H_2_O_2_ (0.1 mM) had no inhibitory effect on the growth of *MRSA* or *E. coli*, whereas the growth of bacteria in the two groups with added materials was inhibited (Fig. 3a, b). In particular, bacterial growth stopped in the Cu SAs/MoS_2_ + H_2_O_2_ group, indicating that, compared with MoS_2_, Cu SAs/MoS_2_ has greater antibacterial activity. The treated bacteria were subsequently subjected to plate culture and counted. The relative survival rates of *MRSA* and *E. coli* in the Cu SAs/MoS_2_ + H_2_O_2_ group were as low as 2.42% and 5.35%, respectively (Fig. 3c, d). Detection via the 2′,7′-dichlorodihydrofluorescein diacetate (DCFH-DA) fluorescent probe revealed that the intracellular ROS level in bacteria in the Cu SAs/MoS_2_ + H_2_O_2_ group was significantly greater than that in the MoS_2_ + H_2_O_2_ group, corresponding to POD-like activity (Figs. 3e, f and S4, S5). In addition, the live‒dead images of bacteria (stained with Syto9/PI) obtained via confocal microscopy were consistent with the survival rates obtained via the plate-counting method (Fig. 3g, h). SEM revealed obvious shrinkage and cracks in the morphology of *MRSA* and *E. coli* in the two material-treated groups, further confirming the antibacterial ability of Cu SAs/MoS_2_ (Fig. 3i). The destruction of the cell wall led to the leakage of a large amount of protein (Fig. 3j). Correspondingly, the permeability of the cell membrane increased significantly after treatment with Cu SAs/MoS_2_ (Fig. 3k). Moreover, analysis via a malondialdehyde (MDA) detection kit revealed that the level of lipid peroxidation increased significantly after treatment with Cu SAs/MoS_2_, which was due to the large amount of ROS that induced lipid peroxidation (Fig. 3l). To explore the antibacterial effects of different concentrations of Cu SAs/MoS_2_, the effects of different concentrations ranging from 0 to 150 μg mL^−1^ on *MRSA* and *E. coli* were detected via the plate-culture method. Cu SAs/MoS_2_ effectively killed these two types of bacteria at a concentration of 150 μg mL^−1^ (Fig. S6a, b). The above results indicate that Cu SAs/MoS_2_, which has enhanced enzyme-like activity, has great potential for use in efficient antibacterial applications.

### Mechanism of the In Vitro Antibacterial Effect of Cu SAs/MoS_2_

**Fig. 4:**
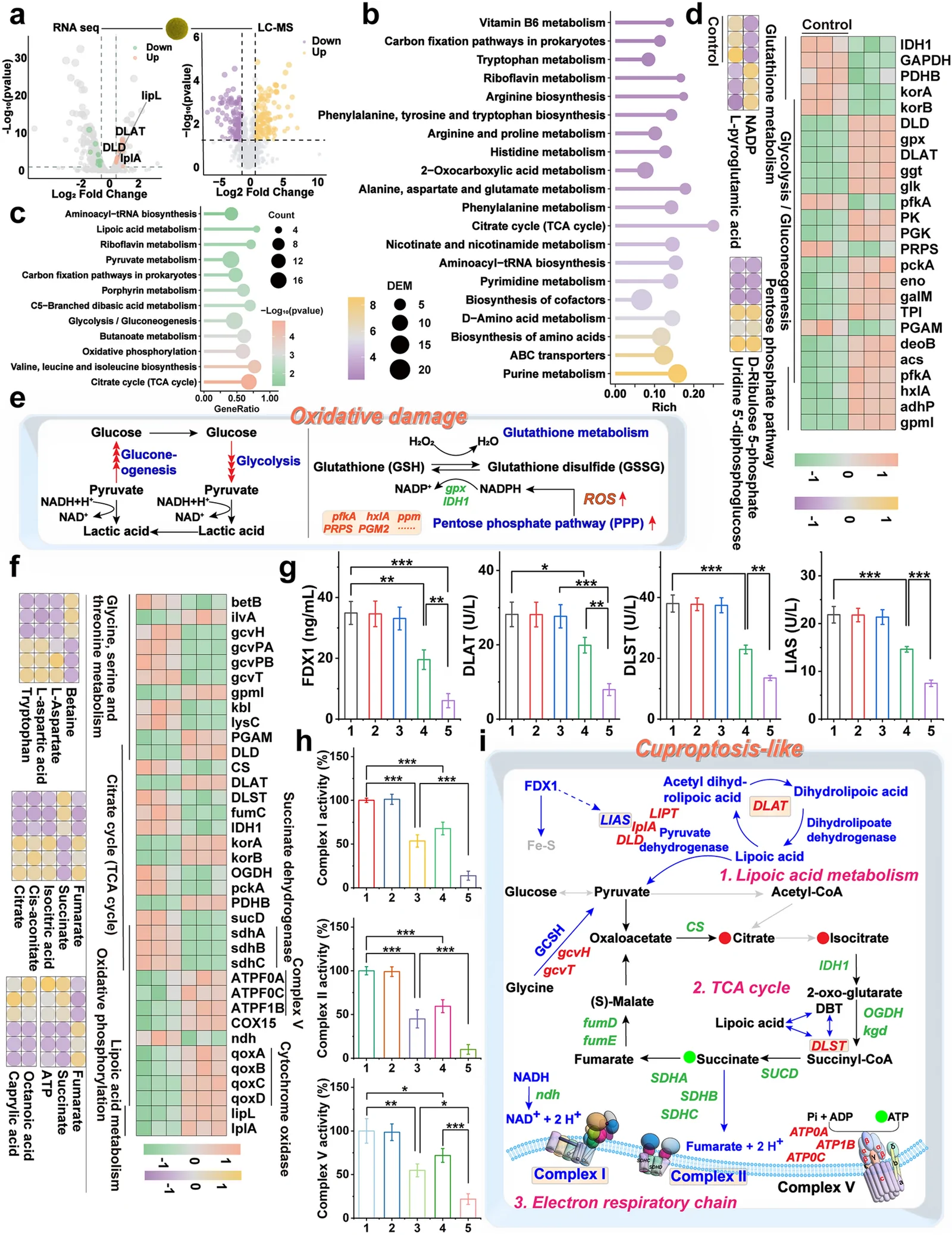
Oxidative damage and cuproptosis-like death induced by Cu SAs/MoS_2_ in drug-resistant bacteria. **a** Volcano maps for prokaryotic transcriptomics and metabolomics. KEGG enrichment analysis of the **b** prokaryotic transcriptome and **c** metabolomics. **d** Heatmap of the expression of genes and metabolites associated with oxidative damage. **e** Schematic of the oxidative damage caused by Cu SAs/MoS_2_. **f** Heatmap of the expression of genes and metabolites associated with cuproptosis-like death. **g** Expression of four important indicators of cuproptosis-like death after treatment.** h** Activity of electron respiratory chain complex I, complex II, and complex V. **i** Schematic of cuproptosis-like death. *Note*: 1: Control; 2: H_2_O_2_; 3: MoS_2_ + H_2_O_2_ + TTM; 4: Cu SAs/MoS_2_ + H_2_O_2_ + VC; 5: Cu SAs/MoS_2_ + H_2_O_2_

To further explore the potential antibacterial mechanism of Cu SAs/MoS_2_ against *MRSA*, a combined analysis method of prokaryotic transcriptomics and metabolomics was employed. A total of 502 differentially expressed genes (DEGs) (224 upregulated and 278 downregulated) and 383 differentially expressed metabolites (181 upregulated and 202 downregulated) were identified by analyzing the combined omics data from the Cu SAs/MoS_2_ and control groups (Figs. 4a and S7, S8). These DEGs and differentially expressed metabolites were significantly enriched in the Kyoto Encyclopedia of Genes and Genomes (KEGG) pathways, which mainly involve energy metabolism, amino acid biosynthesis and metabolism, carbon fixation and metabolism, lipid and cofactor metabolism, etc. (Fig. 4b, c). The bubble chart generated by the KEGG pathway enrichment analysis revealed that multiple pathways related to oxidative damage were significantly enriched in the Cu SAs/MoS_2_ treatment group (Fig. 4d). In the treatment group, bacteria were exposed to a high concentration of ROS, resulting in a significant increase in ROS levels and triggering extensive oxidative damage, including protein, lipid, and energy metabolism. To cope with oxidative stress, bacteria regulate energy metabolism by enhancing glycolysis, lactate production, and gluconeogenesis to maintain metabolic balance. Specifically, in the sugar degradation/gluconeogenesis pathway, key genes such as *PFKA*, *PGK*, *GGT*, *PCK*, and *DEOB* were significantly upregulated, indicating that bacteria enhanced these pathways to cope with oxidative damage (Fig. 4e). In addition, in the pentose phosphate pathway, the genes *hxlA*, *adhP*, and *gpml* were significantly upregulated, which increased the production of D-xylulose 5-phosphate and uridine 5′-diphosphate glucose, supporting the antioxidant function of bacteria. However, despite these measures taken by bacteria, glutathione metabolism, one of the core mechanisms of redox balance, is significantly affected. The expression of key genes such as *IDH1*, *PDHB*, and *GAPDH* was significantly downregulated, and the level of NADP decreased, indicating that a large amount of ROS severely disrupted the bacterial redox balance system. In brief, although bacteria regulate energy metabolism and enhance antioxidant pathways by themselves, this is not sufficient to cope with oxidative stress, and their core glutathione metabolism system is severely disrupted, thus exacerbating oxidative damage.

Notably, the KEGG and heatmap results of both prokaryotic transcriptomics and metabolomics revealed significant enrichment in the TCA cycle, which is closely related to the occurrence of cuproptosis-like death (Fig. 4f). Cuproptosis-like death is a form of programmed bacterial death induced by the accumulation of copper ions (Cu^2+^). The continuous release of Cu^2+^ from Cu SAs/MoS_2_ was detected via ICP over a period of 72 h at pH 5.0 (Fig. S9). To investigate the impacts of oxidative damage and cuproptosis-like death induced by Cu SAs/MoS_2_ on bacterial death, antioxidants (ascorbic acid, VC) and cuproptosis-like death inhibitors (tetrathiomolybdate, TTM) were used for evaluation. VC can directly scavenge various free radicals (such as superoxide anions and hydroxyl radicals), thereby protecting cells from oxidative stress. On the other hand, TTM can bind to Cu^2+^ to form a stable complex, preventing Cu^2+^ from entering cells and binding to lipoylated proteins, thus reducing oxidative stress. During cuproptosis-like death, DLAT, DLST, and LIAS are important markers. After treatment with Cu SAs/MoS_2_ + VC, enzyme-linked immunosorbent assay (ELISA) analysis revealed that the protein expression levels of DLAT, DLST, and LIAS in bacteria were significantly reduced. When Cu SAs/MoS_2_ + TTM was added, the expression levels of these three markers did not significantly differ from those in the control group, indicating that the reduction in their expression was induced mainly by Cu^2+^ (Fig. 4g). Moreover, when neither VC nor TTM was added to the treatment group, the expression levels of the three markers were lower than those in the Cu SAs/MoS_2_ + VC group. These findings suggest that oxidative damage impairs the function of copper ion transport proteins, leading to abnormal intracellular accumulation of Cu^2+^, which in turn enhances the occurrence of cuproptosis-like death and exacerbates bacterial death. Furthermore, although the protein expression levels of DLAT, DLST, and LIAS decreased, the expression levels of the corresponding genes (*DLAT*, *DLST*, *DLD*, *lplA*, *LIPT*) increased in the sequencing analysis. This may be because cuproptosis-like death leads to an increase in the oligomerization of lipoylated proteins, and bacteria upregulate gene expression to compensate for this decrease in protein levels, which is a potential adaptive or repair mechanism. The hallmark of cuproptosis is the oligomerization of lipoylated proteins and the formation of insoluble aggregates. ELISA results can reflect only a decrease in total protein levels and cannot capture this aggregation event. Unfortunately, there are currently no DLAT/DLST antibodies available for western blot validation in bacteria. The development and screening of specific prokaryotic antibodies will be a key step in advancing research on cuproptosis-like death. In addition, Cu^2+^ binds to the iron‒sulfur cluster in ferredoxin 1 (FDX1), disrupting its structure and stability, resulting in impaired FDX1 function and the production of more toxic Cu^+^ (Fig. 4g). Impaired FDX1 function interferes with its role in the electron transport chain, reducing the utilization of intracellular NADPH and the biosynthesis of iron‒sulfur clusters, thereby affecting the normal operation of the TCA cycle and the electron transport chain. In this study, by comparing three treatment methods, namely, oxidative damage (Cu SAs/MoS_2_ + TTM), cuproptosis-like death (Cu SAs/MoS_2_ + VC), and combined action (Cu SAs/MoS_2_), the Cu SAs/MoS_2_ group exhibited a greater ability to inhibit the enzymatic activities of electron respiratory chain complex I, complex II, and complex V through a dual mechanism (Fig. 4h). This enhanced dual-mechanism effect is due to the synergistic action of Cu^2+^-induced cuproptosis-like death and oxidative stress, which in turn leads to more significant inhibition of cell function (Fig. 4i). In brief, Cu SAs/MoS_2_ significantly enhances the antibacterial effect against bacteria through the synergistic action of cuproptosis-like death and oxidative stress.

### Regulation of Bacterial Drug Resistance Development by Cu SAs/MoS_2_

**Fig. 5:**
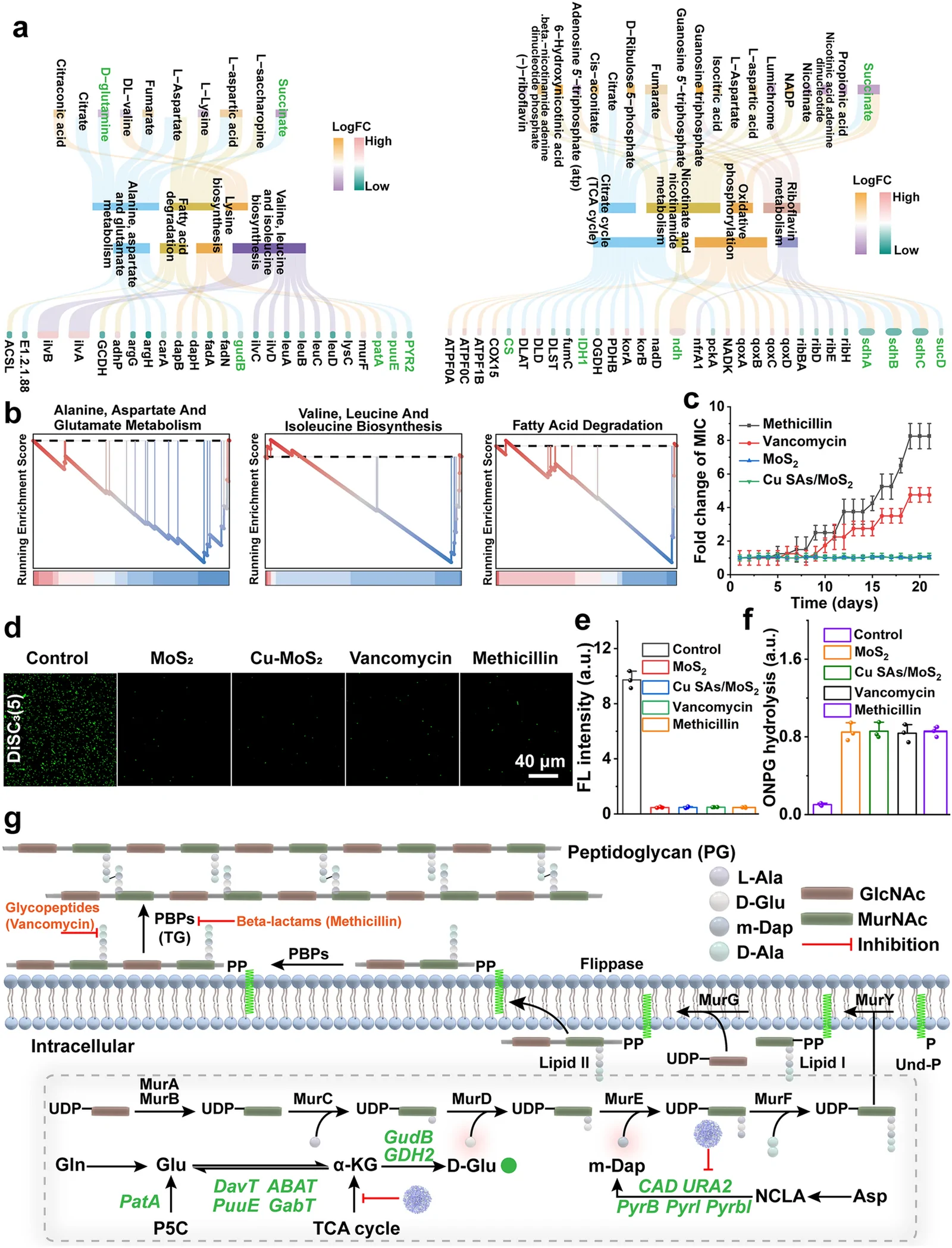
Studies on the mechanism by which Cu SAs/MoS_2_ retard the development of drug resistance in bacteria. **a** Cell wall synthesis- and energy metabolism-related pathway Sankey diagrams. **b** KEGG enrichment in GSEA. **c** Evolution of *MRSA* resistance to MoS_2_, Cu SAs/MoS_2_, vancomycin, and methicillin after 21 days of incubation in liquid medium. **d** DiSC_3_(5) test showing the effects of materials on the outer membrane permeability of *MRSA*. **e** Quantitative analysis of DiSC_3_(5) fluorescence image statistics. **f** ONPG test showing the effects of materials on *the* outer membrane permeability of *MRSA*. **g** Schematic representation of the role of Cu SAs/MoS_2_ in reducing the development of drug resistance

The impact of bacterial resistance on nanozyme antibacterial therapy is highly concerning. For example, the resistance of *MRSA* is achieved by altering the structure of PBPs or producing β-lactamases to degrade antibiotics. Resistance mutations often impose a burden on bacterial growth and metabolism. Therefore, *MRSA* undergoes a series of compensatory mutations to alleviate these negative effects. Compensatory mutations involve multiple factors, including influencing genes related to energy metabolism to maintain energy supply, altering transcriptional regulatory factors to optimize gene expression, adjusting the cell wall synthesis pathway to maintain structural integrity, and enhancing biofilm formation ability to improve survival. A Sankey diagram of the results of the metabolomics and transcriptome sequencing analyses revealed that key pathways involved in energy metabolism, such as the TCA cycle; oxidative phosphorylation; riboflavin metabolism; nicotinate and nicotinamide metabolism; and multiple pathways related to cell wall synthesis, including valine, leucine and isoleucine biosynthesis; alanine, aspartate and glutamate metabolism; fatty acid degradation; and lysine biosynthesis, were significantly enriched after treatment with Cu SAs/MoS_2_ (Fig. 5a). In particular, the KEGG analysis of the gene set enrichment analysis (GSEA) revealed that the metabolic pathways of valine, leucine and isoleucine biosynthesis; alanine, aspartate and glutamate metabolism; and fatty acid degradation were all downregulated (Fig. 5b). These pathways provide essential precursor substances for cell wall construction. These findings indicate that Cu SAs/MoS_2_ not only interferes with the bacterial energy metabolism process through oxidative damage and cuproptosis-like death mechanisms but also directly affects cell wall synthesis-related pathways. After treatment with Cu SAs/MoS_2_, multiple key genes involved in the biosynthetic pathways of alanine, aspartate, and glutamate metabolism, such as *GudB*, *GDH2* (encoding D-glutamate, D-Glu), and *CAD*, *URA2*, *PyrB*, *Pyrl*, and *Pyrbl* (encoding m-diaminopimelic acid, m-DAP), were downregulated, and the levels of two metabolites, D-Glu and m-DAP, were significantly reduced. These two proteins are key components of peptidoglycan cross-linking, ultimately leading to impaired synthesis and cross-linking of peptidoglycan and weakening the integrity of the cell wall. In addition, the inhibition of fatty acid degradation reduces the supply of lipid components to the cell wall, thereby weakening its integrity and function. The downregulation of leucine, isoleucine, and valine biosynthesis limits the supply of branched-chain amino acids required for cell wall protein synthesis. Therefore, the downregulation of these metabolic pathways not only reduces the precursor substances and energy required for cell wall synthesis but also directly affects the synthesis of D-Glu and m-DAP, further weakening the structure and function of the cell wall and increasing bacterial vulnerability to external stress. In brief, Cu SAs/MoS_2_ interferes with the compensatory mechanisms of drug-resistant bacteria, preventing bacteria from effectively maintaining their energy status and cell wall integrity. This not only weakens the adaptability of bacteria in adverse environments but also fundamentally reduces their drug resistance.

The MIC is a core indicator for measuring the scientific value of antibacterial nanozymes. For example, Zhang et al. reported that the MIC of dextran guanidinylated carbon dots was as low as 5 μg mL^−1^, demonstrating excellent antibacterial performance [47]. On this basis, the MICs of MoS_2_ and Cu SAs/MoS_2_, which were 17 and 73 μg mL^−1^, respectively, were evaluated (Fig. S10). Additionally, the risk of Cu SAs/MoS_2_-induced *MRSA* resistance development was further evaluated. Under continuous dosing conditions, after 21 generations of passage, the MICs of vancomycin and methicillin increased by eightfold and fivefold, respectively, with stable emergence of resistant strains. In contrast, the MICs for MoS_2_ and Cu SAs/MoS_2_ remained stable, with no significant resistance observed (Fig. 5c). Intermittent dosing showed that the evolution of resistance to vancomycin and methicillin was delayed but still on the rise, whereas the MICs of MoS_2_ and Cu SAs/MoS_2_ did not increase, suggesting that their antibacterial mechanism is not easily triggered to cause resistance (Fig. S11). Furthermore, the concentration of copper ions in the Cu SAs/MoS_2_ (150 μg mL^−1^) used for treatment was only 0.0745 μg mL^−1^ (Fig. S12). This low copper content of the nanozyme ensures good safety, which is beneficial for in vivo treatment. Next, after the bacteria treated with the four drugs for 20 generations were further treated with Cu SAs/MoS_2_, 3,3’-dipropylthiadicarbocyanine iodide (DiSC_3_(5)) was used to detect the polarization state of the cytoplasmic membrane. Compared with that of the control group, the fluorescence intensity of all four treatment groups decreased significantly, indicating an increased degree of depolarization of the cytoplasmic membrane (Fig. 5d, e). There was no significant difference in fluorescence intensity among the treatment groups, suggesting that treatment with Cu SAs/MoS_2_ was not affected by the development of resistance in the four different types of bacteria. Furthermore, the ortho-nitrophenyl-β-d-galactopyranoside (ONPG) test was used to evaluate membrane permeability. After treatment with Cu SAs/MoS_2_, the absorbance values of the four types of drug-resistant bacteria increased significantly, and the changes in membrane permeability were basically consistent among the four groups (Fig. 5f). These results echo the inferences from the two-omics analyses (Fig. 5g). In brief, Cu SAs/MoS_2_ did not induce the development of bacterial resistance and showed a stable ability to disrupt bacteria with different levels of resistance development, demonstrating its potential for consistent efficacy in dealing with drug-resistant bacteria.

### In Vitro Antibiofilm Activity

**Fig. 6:**
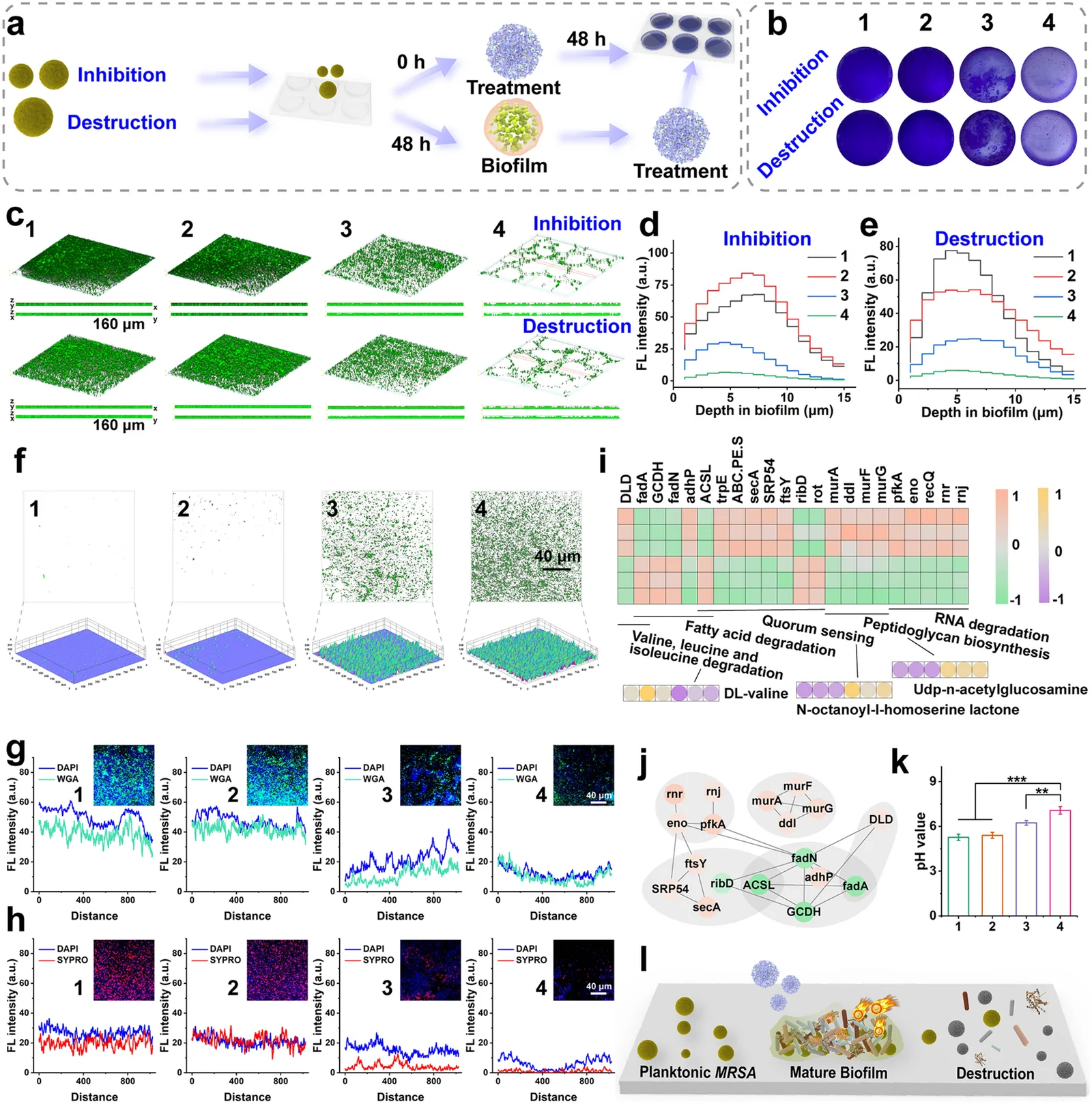
In vitro antibiofilm properties of Cu SAs/MoS_2_. **a** Schematic diagram of Cu SAs/MoS_2_ disruption and the inhibition of *MRSA*-formed biofilm processes. **b** Photographs of disrupted and inhibited biofilms after different treatments were stained with crystal violet. 1: control; 2: H_2_O_2_; 3: MoS_2_ + H_2_O_2_; 4: Cu SAs/MoS_2_ + H_2_O_2_. **c** 3D fluorescence images of inhibited and disrupted biofilms. Fluorescence intensity of **d** inhibition and **e** disruption at different biofilm depths. **f** Fluorescence images of ROS expression. Fluorescence images and coquantitative analysis of **g** protein and **h** polysaccharide expression levels in biofilms. **i** Heatmap of gene expression associated with biofilm synthesis via prokaryotic transcriptome sequencing of *MRSA*.** j** Protein‒protein interaction (PPI) network diagram.** k** Biofilm pH value after treatment. **l** Schematic diagram of a biofilm after disruption

Although Cu SAs/MoS_2_ exhibits excellent antibacterial performance and can effectively reduce the development of drug resistance in vitro, biofilms significantly weaken the antibacterial effect of Cu SAs/MoS_2_ and accelerate the evolution of drug resistance by forming a physical barrier and altering the microenvironment. Here, we investigated the different effects of Cu SAs/MoS_2_ before and after biofilm formation, including its ability to inhibit biofilm formation or disrupt the already formed biofilm (Fig. 6a). In the control wells, crystal violet staining revealed an intact biofilm structure. However, treatment with MoS_2_ and Cu SAs/MoS_2_ significantly promoted biofilm disruption and inhibition. Compared with those in the biofilm disruption group, the biofilms in the MoS_2_ and Cu SAs/MoS_2_ groups presented a more discrete morphology during biofilm inhibition (Fig. 6b). Moreover, the biofilm clearance ability of the Cu SAs/MoS_2_ group was greater than that of the MoS_2_ group in both tests. By measuring the OD_595_ value with a microplate reader and performing calculations, the relative inhibition rate of Cu SAs/MoS_2_ on the biofilm was 91.88%, and the relative disruption rate was 85.81% (Figs. S13 and S14). By labeling live bacteria with PI stain and constructing 3D fluorescent biofilm images, the biofilms in the inhibition and disruption groups presented weak green fluorescence and a discrete morphology, which was consistent with the results of the crystal violet experiment (Fig. 6c). Fluorescence quantitative analysis of the 15-μm-thick biofilm revealed that the fluorescence intensity of the deep-layer biofilm decreased significantly, indicating that the biofilm was severely damaged (Fig. 6d, e). Subsequently, detection with the DCFH-DA probe revealed that the biofilms treated with MoS_2_ and Cu SAs/MoS_2_ produced a large amount of ROS, which was an important reason for biofilm disruption (Fig. 6f). SEM images further revealed the microscopic structure of the biofilm disruption after Cu SAs/MoS_2_ treatment (Fig. S15). In addition, biofilm disruption is usually accompanied by the release of polysaccharides (stained with wheat germ agglutinin (WGA)) and proteins (stained with SYPRO) in the extracellular polymeric substances (EPS), which are signs of biofilm structure interference and decomposition. In the MoS_2_ and Cu SAs/MoS_2_ groups, the EPS components of the biofilm were obviously degraded and released, which further confirmed the destruction of the biofilm structure (Fig. 6g, h). In particular, the Cu SAs/MoS_2_ treatment group presented greater EPS release, indicating its stronger biofilm-disrupting ability. Moreover, the pH of the biofilm supernatant of the Cu SAs/MoS_2_ group returned to neutral (Fig. 6k). Notably, according to the two-omics sequencing results, multiple key pathways closely related to biofilm formation, stability, and function, such as RNA degradation, quorum sensing, peptidoglycan biosynthesis, fatty acid degradation, and valine, leucine, and isoleucine degradation, were significantly enriched (Fig. 6i). Among them, genes related to quorum sensing, such as *trpE*, *secA*, *SRP54*, and *ftsY*, were significantly downregulated, which inhibited the generation and transmission of signal molecules, resulting in the inability of bacteria to effectively coordinate group behaviors. This disrupted the formation and stability of the biofilm and affected the communication and cooperation of bacteria in a high-density population environment (Fig. 6j). In addition, at different growth stages of the biofilm (such as initial attachment, maturation, and dispersion), inhibiting RNA degradation, peptidoglycan biosynthesis, fatty acid degradation, and branched-chain amino acid degradation hinders gene expression regulation, structural support, energy supply, and the provision of metabolic precursors, thereby effectively killing bacteria and weakening the adaptability and stability of the biofilm. These results indicate that Cu SAs/MoS_2_ leads to consequences such as hindered biofilm formation, an unstable structure, and altered metabolic activity (Fig. 6l). In brief, Cu SAs/MoS_2_ significantly disrupts the structure and function of biofilms, inhibits biofilm formation, weakens biofilm stability by generating ROS and inhibiting key metabolic pathways, demonstrating strong antibacterial and drug resistance development-inhibiting abilities.

### In Vivo Antibacterial Activity and Wound Healing Mechanisms

Considering that biosafety is a critical prerequisite for the biomedical application of nanomaterials in vivo, we conducted cell viability assays with HaCaT, HUVEC, RAW 264.7, and L929 cells and hemolysis tests to evaluate the safety of Cu SAs/MoS_2_ in the treatment of traumatic infections. The experimental results showed that Cu SAs/MoS_2_ exhibited good biocompatibility in all the tested cell lines. The cell viability remained above 80% in the concentration range of 50 to 500 μg mL^−1^, meeting the ISO 10993–5 standard. Even at a high concentration of 500 μg mL^−1^, the cell activity did not decrease significantly, indicating that Cu SAs/MoS_2_ has high safety and biocompatibility (Fig. S16). Moreover, the hemolysis rate of red blood cells caused by Cu SAs/MoS_2_ was less than 5% at a concentration of 150 μg mL^−1^, indicating excellent blood compatibility (Fig. S17). These results together confirm the potential of Cu SAs/MoS_2_ for safe application in the treatment of wound infections.

Wounds infected with bacteria often develop a purulent inflammatory microenvironment. To verify its catalytic activity in purulent inflammation, an in vitro inflammatory microenvironment simulation system and a subcutaneous abscess mouse model were established simultaneously to assess the functional stability of the catalytic system in the pus environment. In vitro experiments revealed significant increases in the levels of IL-1β and TNF-α, confirming the successful construction of the inflammatory microenvironment (Fig. S18). In PBS and the inflammatory microenvironment, the POD-like and GSH-Px-like activities of Cu SAs/MoS_2_ remained stable (Fig. S19). Next, a subcutaneous abscess mouse model was successfully constructed to further evaluate the antibacterial effect of Cu SAs/MoS_2_ in vivo (Fig. S20a). On the seventh day of treatment, the wound had decreased in swelling, and the residual bacterial count in the skin tissue had also decreased (Fig. S20b, c). H&E staining revealed that the epidermis in the treatment group had basically re-epithelialized, with reduced inflammatory cell infiltration in the dermis, disappearance of abscess cavities, and an abundance of new capillaries. Masson staining revealed an increase in the area of blue-stained collagen fibers, which were densely arranged and parallel to the epidermis, indicating the formation of mature granulation tissue. Gemsa staining further confirmed that no bacterial colonies were observed in the subcutaneous tissue of the treatment group (Fig. S20d). In brief, the Cu SAs/MoS_2_ constructed in this work can maintain high catalytic activity and therapeutic efficacy even in a purulent inflammatory environment.

**Fig. 7:**
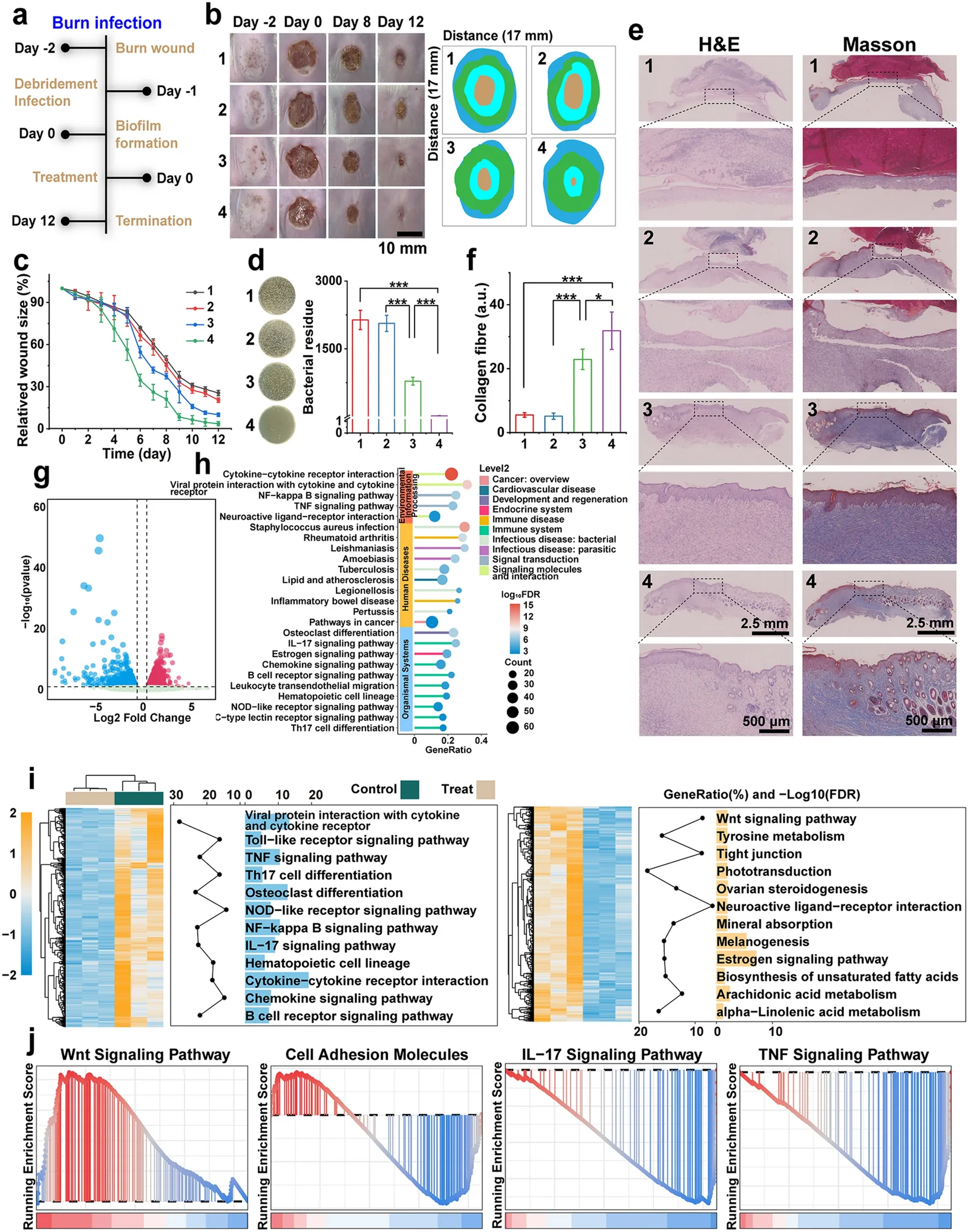
Cu SAs/MoS_2_ exhibits antibacterial efficacy in vivo and improves wound healing outcomes.** a** Schematic diagram of the construction of the burn *MRSA*-infected wound model and its treatment. **b** Photographs of the wounds and **c** relative wound healing rates at 12 days. **d** Plate photographs of bacterial residues in skin tissue on the sixth day and relative bacterial survival. **e** H&E and Masson staining images of the corresponding wounds after different treatments. **f** Semiquantitative results of collagen fibers in Masson images. **g** Volcano map of significantly upregulated (red) and downregulated (blue) genes.** h** GO enrichment analysis.** i** Heatmap and KEGG pathway enrichment analysis. **j** KEGG enrichment in GSEA

A burn wound model infected with *MRSA* was further used to evaluate the in vivo antibacterial ability of Cu SAs/MoS_2_ (Fig. 7a). After treatment with Cu SAs/MoS_2_, the wound healing photographs taken within the 12-day monitoring period revealed the best healing effect (Fig. 7b). On the 12th day, the relative wound size in the Cu SAs/MoS_2_ group was 3.68%, which was significantly greater than that in the control group (25.47%), H_2_O_2_ group (20.44%), and MoS_2_ group (10.07%) (Fig. 7c). On the 6th day after treatment, the residual bacteria at the wound site were evaluated via plate culture. Compared with those in the control group, the residual bacteria in the Cu SAs/MoS_2_ group were reduced by more than 95%, indicating the high-efficiency antibacterial performance of Cu SAs/MoS_2_ (Fig. 7d). Histological examination revealed that, in H&E-stained sections, the Cu SAs/MoS_2_ treatment group presented significantly improved tissue structure, with distinct stratification of the epidermis and dermis. In the Masson’s trichrome-stained sections, the collagen fibers appeared uniformly blue and were regularly arranged, indicating good and orderly deposition of collagen fibers, which demonstrated enhanced tissue repair and regeneration (Fig. 7e, f).

In addition, burn wound models infected with *E. coli* were further used to evaluate the in vivo antibacterial ability of Cu SAs/MoS_2_ (Fig. S21a). The wounds in the Cu SAs/MoS_2_ group also showed significant and sustained advantages in terms of wound healing on day 12 (Fig. S21b, c). The bacterial plate results revealed that bacterial residues in the wounds of the treatment groups were significantly lower than those in the wounds of the PBS control groups, which was consistent with the findings in the *MRSA* infection model (Fig. S21d). H&E and Masson staining revealed that inflammatory cell infiltration, collagen deposition, and re-epithelialization were lower in the treatment group than in the control group (Fig. S21e). However, further validation is still needed in future studies of different gram-negative bacterial strains (especially clinically resistant strains).

To further evaluate the wound healing mechanism, eukaryotic transcriptome sequencing analysis was performed on skin tissues. The volcano plot revealed that there were 661 upregulated genes (in red) and 673 downregulated genes (in blue) in the skin samples (Fig. 7g). GO enrichment analysis indicated that Cu SAs/MoS_2_ was significantly enriched in multiple biological functions, such as the inflammatory response, immune system, and signal transduction (Fig. 7h). KEGG enrichment analysis and heatmaps revealed that most inflammation-related pathways (e.g., the IL-17 signaling pathway and TNF signaling pathway) were downregulated, whereas pathways related to cell proliferation and the immune system (e.g., the Wnt signaling pathway and estrogen signaling pathway) were upregulated (Fig. 7i). These findings suggest that the skin has transitioned from the inflammatory response phase to the phases of cell proliferation and immune system activation. In addition, the GSEA plot, which presents the enrichment scores and normalized enrichment scores for each gene set, further confirmed the results of the KEGG enrichment analysis (Fig. 7j).

**Fig. 8:**
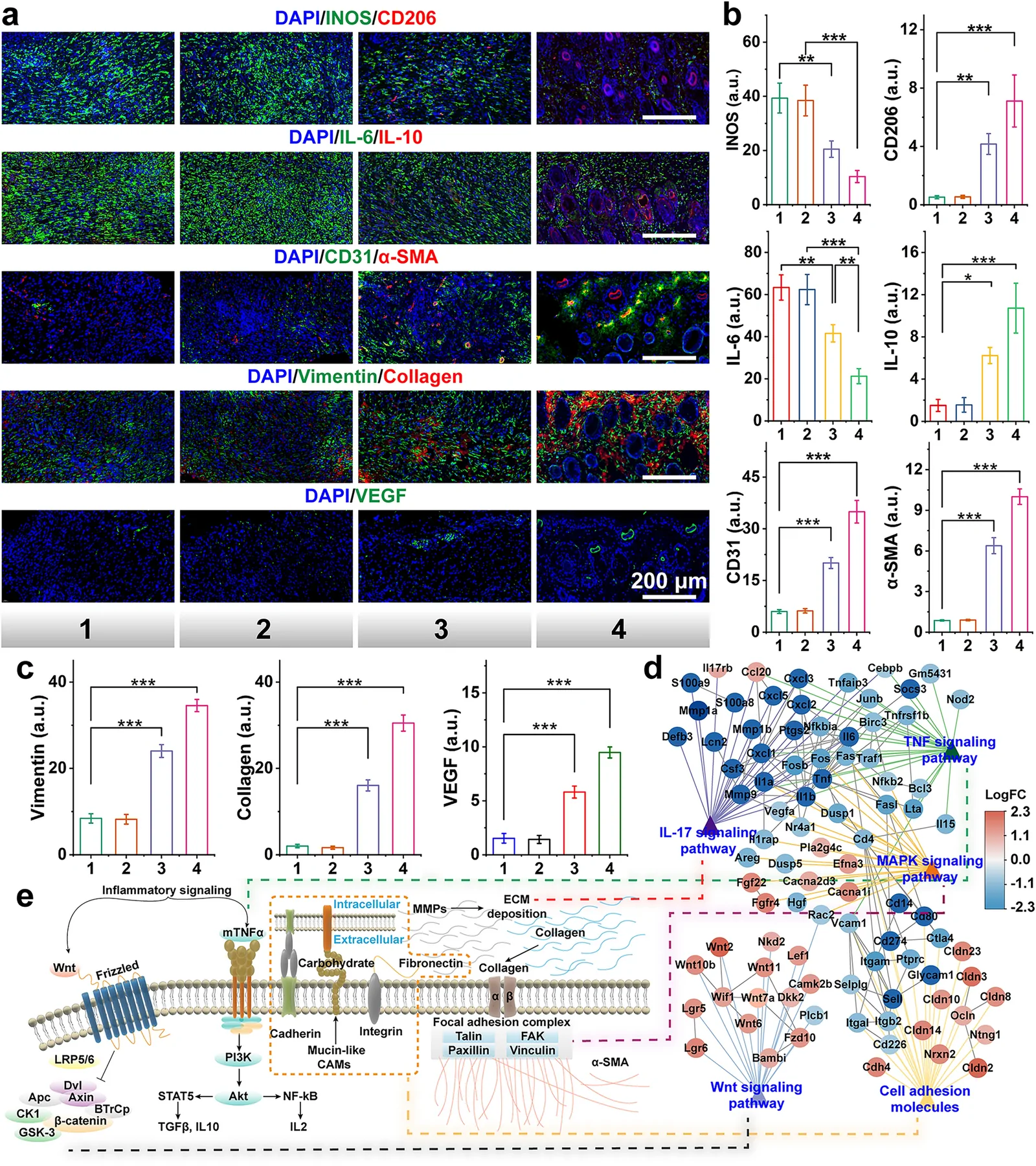
Study of the promotion of wound healing by Cu SAs/MoS_2_.** a** Fluorescence images and **b**, **c** quantitative analysis of indicators related to the promotion of wound healing. **d** PPI network diagram of Cu SAs/MoS_2_ regulatory pathways and **e** schematic of wound healing. 1: control; 2: H_2_O_2_; 3: MoS_2_ + H_2_O_2_; 4: Cu SAs/MoS_2_ + H_2_O_2_

Eukaryotic transcriptome sequencing analysis revealed inflammation resolution and tissue repair during the wound healing process. On the 12th day, fluorescence staining of biomarkers in mouse skin tissues revealed that treatment with Cu SAs/MoS_2_ effectively alleviated the inflammatory response in the skin tissue (decreased levels of iNOS and IL-6) while enhancing the anti-inflammatory and tissue repair processes (increased levels of CD206 and IL-10) (Fig. 8a, b). These findings indicate a polarization shift of macrophages from the proinflammatory M1 type to the anti-inflammatory and repair-promoting M2 type, which promotes inflammation resolution and tissue healing, confirming the positive role of Cu SAs/MoS_2_ in promoting skin repair. In addition, the elevated expression of CD31 and VEGF reflects enhanced angiogenic activity (Fig. 8a–c). The elevated expression of α-SMA indicates increased myofibroblast activity, which is a sign of mature tissue repair and a more stable tissue structure. Moreover, the increased expression of vimentin and collagen reflects enhanced mesenchymal cell activity and increased extracellular matrix synthesis, which promotes tissue remodeling and repair and strengthens structural support and integrity. Additionally, these results coincide with the trend of collagen fiber expression presented by the Masson images. In brief, treatment with Cu SAs/MoS_2_ regulated metabolic pathways related to the inflammatory response, angiogenesis, extracellular matrix synthesis, and tissue remodeling, effectively promoting skin tissue repair and structural stability (Fig. 8d, e).

Notably, the in vivo biosafety results revealed that H&E staining of the heart, liver, spleen, lungs, and kidneys revealed no obvious abnormalities. The structures of all the organs were normal, without lesions such as inflammatory cell infiltration, fibrosis, necrosis, or calcification (Fig. S22a). Routine blood examinations revealed that the WBC, RBC, HGB, PLT, and other related indicators were within the normal ranges. These findings suggest that the treatment process did not have an adverse effect on the blood system and that there were no obvious signs of infection, inflammation, or anemia in the body, indicating good overall health status (Fig. S22b). Blood biochemical results revealed that the levels of CRE, ALT, AST, and BUN were all within the normal ranges, indicating that the treatment had no adverse effects on liver or kidney functions and that the in vivo metabolic state was stable (Fig. S22b). On the second and twelfth days after treatment, the copper ion levels in the Cu SAs/MoS_2_ composite remained consistent with those in the control group, with no significant differences observed (Fig. S23). This result indicated that the copper ions released by Cu SAs/MoS_2_ could be effectively cleared or redistributed within two days without accumulating in the blood or major organs, thereby avoiding the risk of copper-related systemic toxicity. In brief, the treatment demonstrated good safety for various organs and systems in the animals, and no significant adverse reactions were observed.

## Conclusion

In this study, a multifunctional nanozyme (Cu SAs/MoS_2_) was successfully developed for the treatment of burn-infected wounds. The POD-like and GSH-Px-like activities of Cu SAs/MoS_2_ were significantly enhanced by loading Cu SAs onto MoS_2_ in an atomically uniform manner. The abundant release of ROS from Cu SAs/MoS_2_ inhibited multiple metabolic pathways of bacteria, demonstrating potent antibacterial capabilities. Concurrently, oxidative damage led to a large influx of Cu^2+^ into the bacteria, inducing programmed bacterial cuproptosis-like death. Cu SAs/MoS_2_ disrupted the compensatory pathways of drug-resistant bacteria by inhibiting their energy metabolism and cell wall synthesis, which not only effectively inhibited the survival of drug-resistant bacteria but also limited the development of bacterial resistance. The in vivo results showed that Cu SAs/MoS_2_ significantly alleviated inflammatory responses while promoting angiogenesis, cell proliferation, and tissue remodeling, comprehensively improving the healing process of burn wounds. This work develops an idea in the field of nanozyme design and provides an innovative therapeutic strategy to address the global problem of antibiotic resistance, which is expected to lead to a breakthrough in the treatment of drug-resistant infections.

## Supplementary Information

Below is the link to the electronic supplementary material.

Supplementary file 1 (DOCX 6739 kb)
