# Supplementary material for: Copper Single-Atoms Loaded on Molybdenum Disulphide Drive Bacterial Cuproptosis-Like Death and Interrupt Drug-Resistance Compensation Pathways
Source: Nanomicro Lett. 2026 Jan 11;18:111. doi: 10.1007/s40820-025-01955-2 (PMC12790557; doi:10.1007/s40820-025-01955-2)
Supplement: Supplementary file 1 — (DOCX 6739 kb) [file 40820_2025_1955_MOESM1_ESM.docx]

Supporting Information for

**Copper Single-Atoms Loaded on Molybdenum Disulphide Drive Bacterial Cuproptosis-Like Death and Interrupt Drug-Resistance Compensation Pathways**

Wenqi Wang^1, 2^, Xiaolong Wei^3^, Bolong Xu^4^, Hengshuo Gui^3^, Yan Yan^5^*, Huiyu Liu^4^*, Xianwen Wang^1, 3^*

^1^ College and Hospital of Stomatology, Anhui Medical University, Key Lab of Oral Diseases Research of Anhui Province, Hefei 230032, P. R. China

^2^ School of Pharmaceutical Sciences, Anhui Medical University, Hefei 230032, P. R. China

^3^ School of Biomedical Engineering, Anhui Medical University, Hefei 230032, P. R. China

^4^ Beijing Advanced Innovation Center for Soft Matter Science and Engineering, State Key Laboratory of Organic‒Inorganic Composites, Bionanomaterials and Translational Engineering Laboratory, Beijing Key Laboratory of Bioprocess, Beijing Laboratory of Biomedical Materials, Beijing University of Chemical Technology, Beijing 100029, P. R. China

^5^ School of Chemistry & Chemical Engineering, Anhui University of Technology, Ma’anshan, Anhui 243002, P. R. China

*****Corresponding authors. E-mail: [xianwenwang@ahmu.edu.cn](mailto:xianwenwang@ahmu.edu.cn) (Xianwen Wang); [liuhy@mail.buct.edu.cn](mailto:liuhy@mail.buct.edu.cn) (Huiyu Liu); [yanyan@ahut.edu.cn](mailto:yanyan@ahut.edu.cn) (Yan Yan)

**Supplementary Figures and Table**


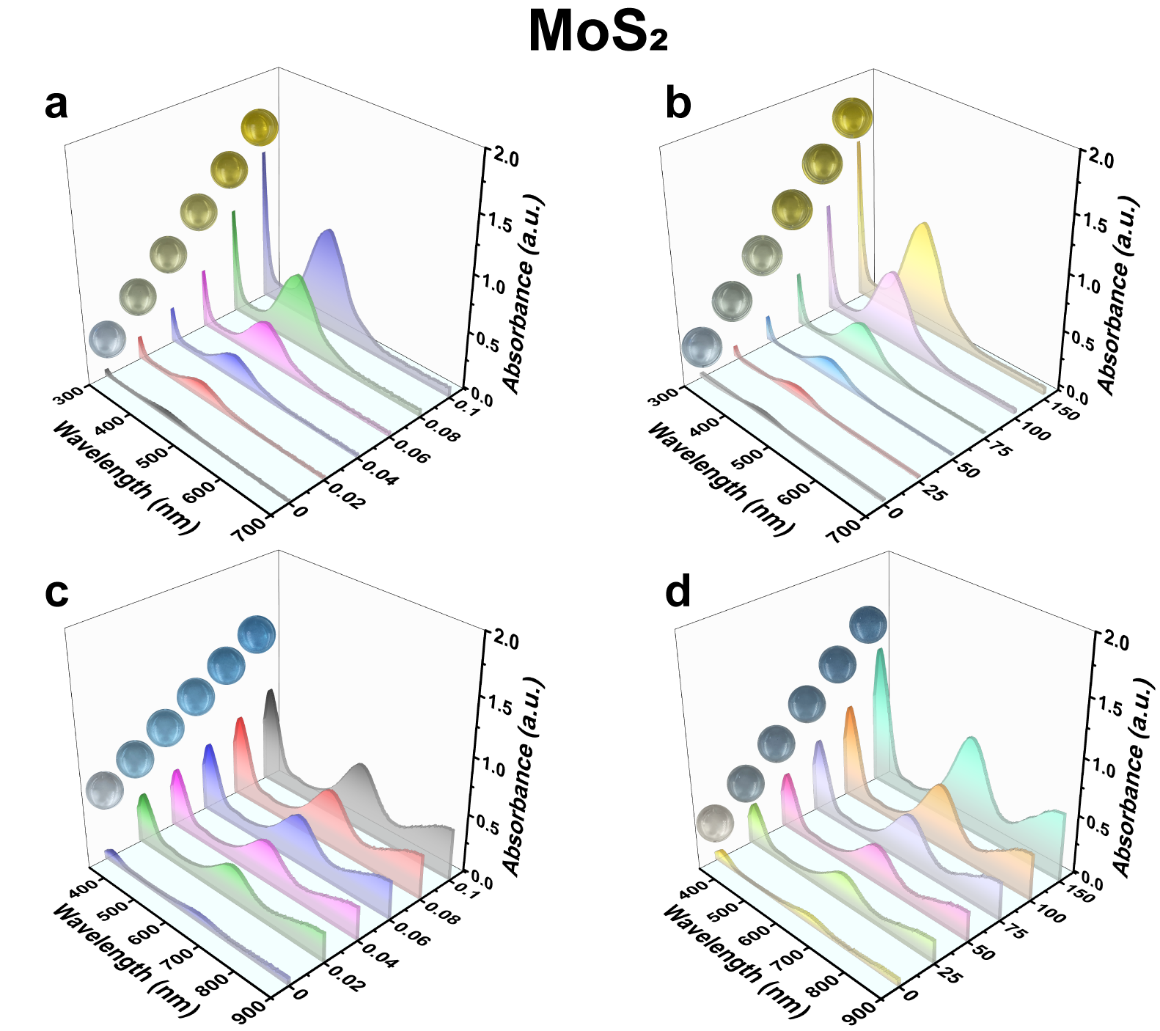


**Fig. S1** POD-like activity of MoS_2_ at different H_2_O_2_ concentrations using **a** OPD and **b** TMB. POD-like activity at different MoS_2_ concentrations using **c** OPD and **d** TMB


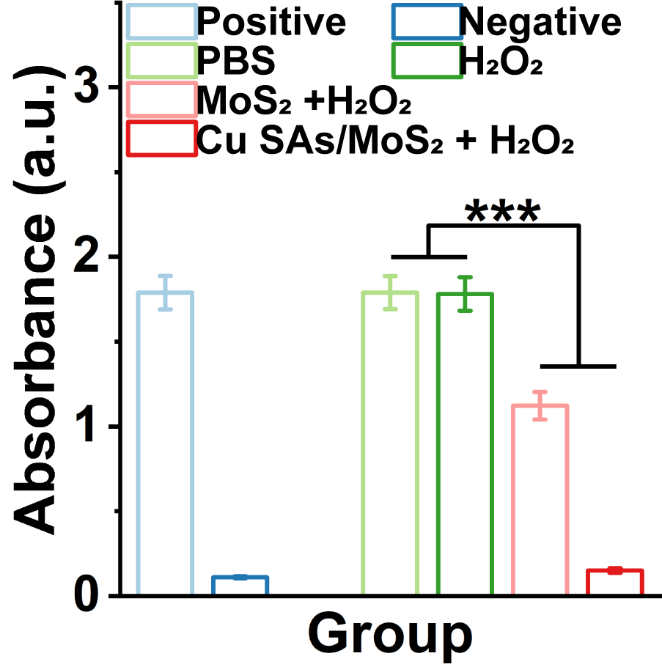


**Fig. S2** Quantification of the GSH consumption capacity after different treatments


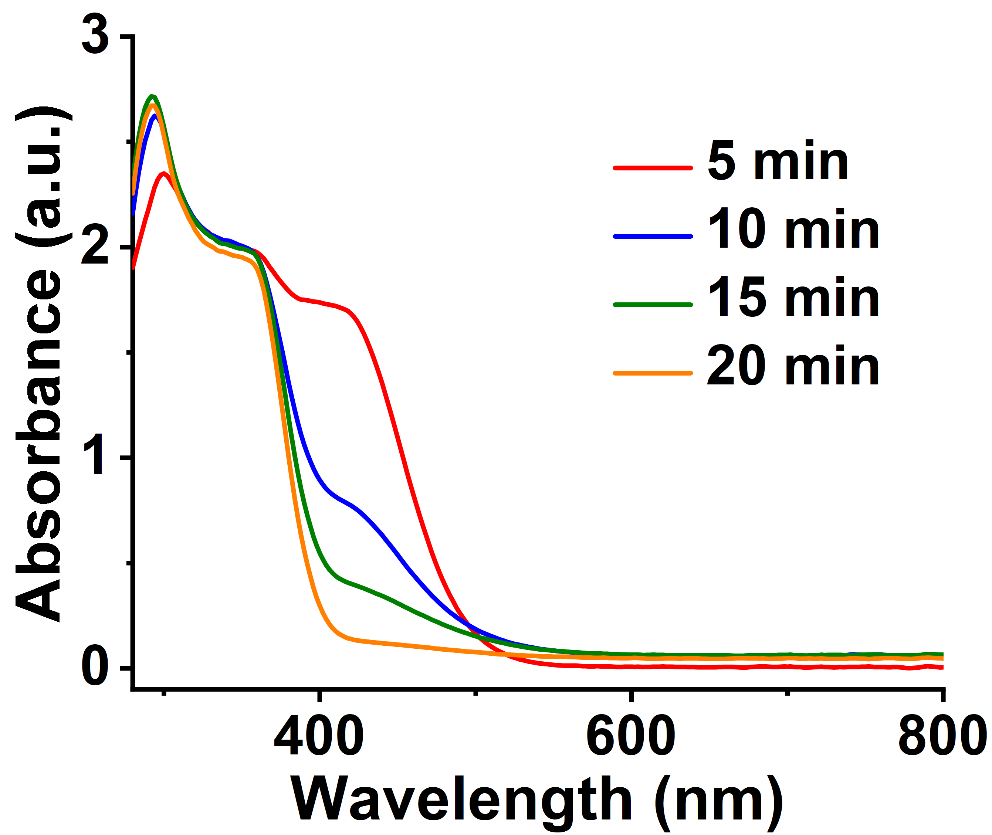


**Fig. S3** Time-dependent GSH depletion of Cu SAs/MoS_2_ at a concentration of 150 μg/mL using the DTNB probe


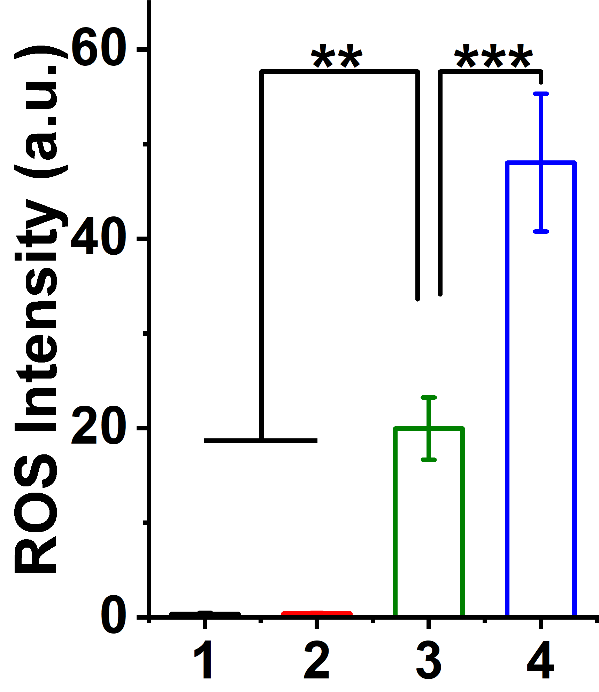


**Fig. S4** Quantitative analysis of ROS in *MRSA* subjected to different treatments via DCFH-DA


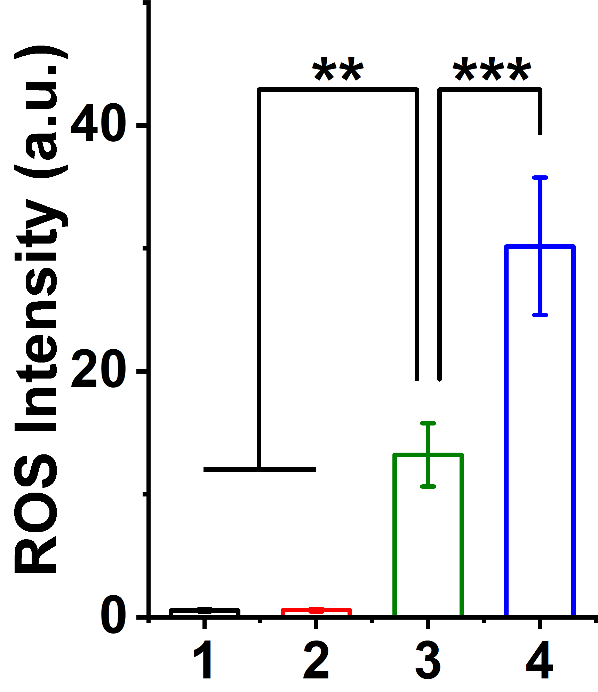


**Fig. S5** Quantitative analysis of ROS in *E. coli* subjected to different treatments via DCFH-DA


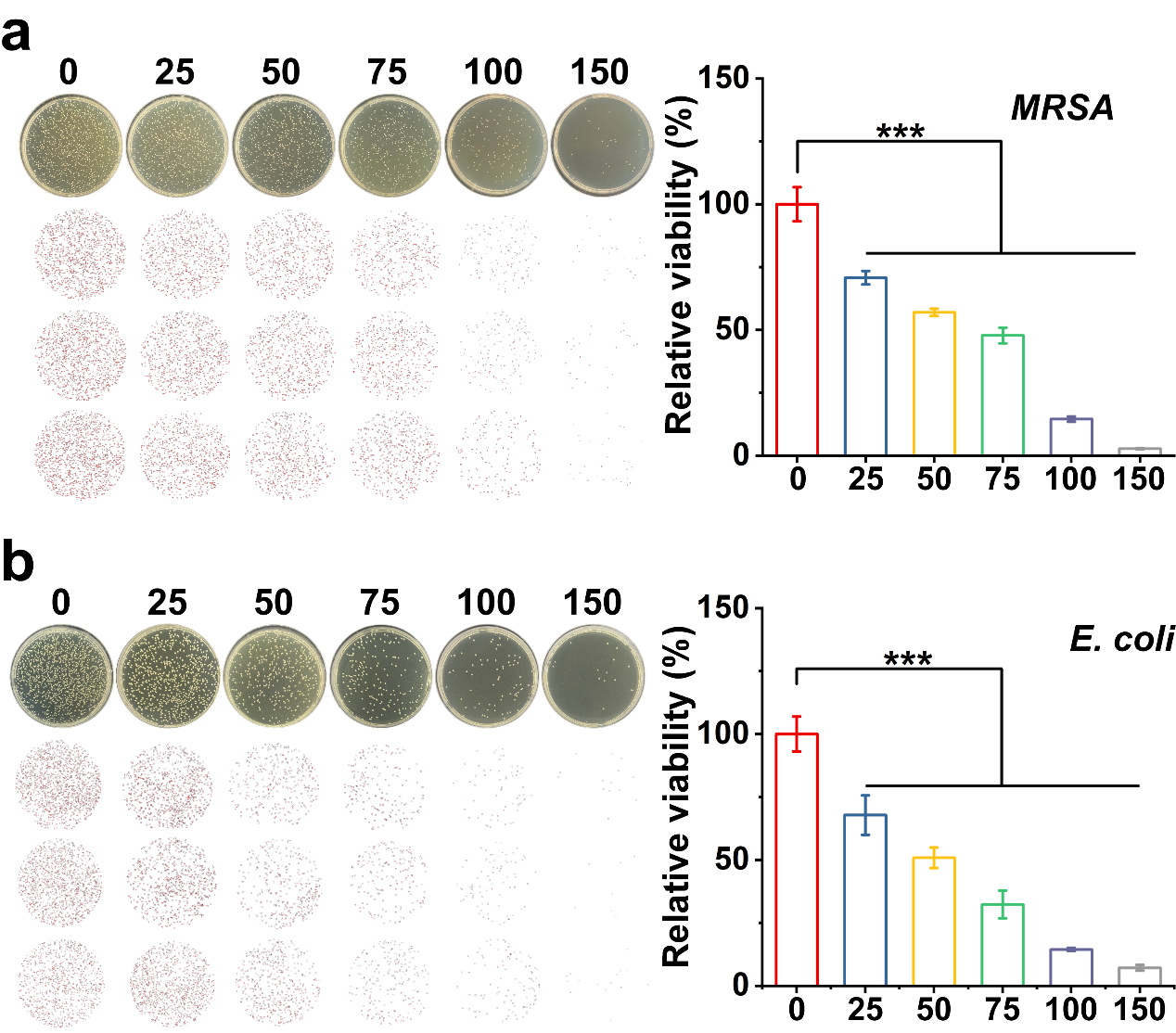


**Fig. S6** Plate photographs and corresponding quantitative survival rates after treatment of **a** *MRSA* and **b** *E. coli* with different Cu SAs/MoS_2_ concentrations


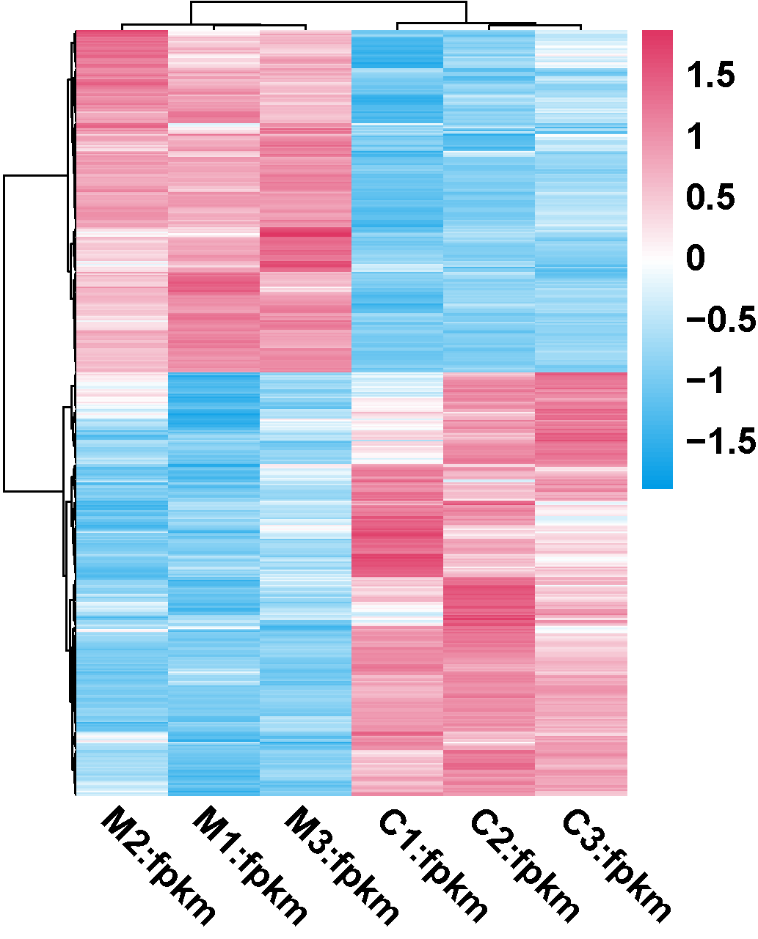


**Fig. S7** Heatmap of differentially expressed genes identified via prokaryotic transcriptomics


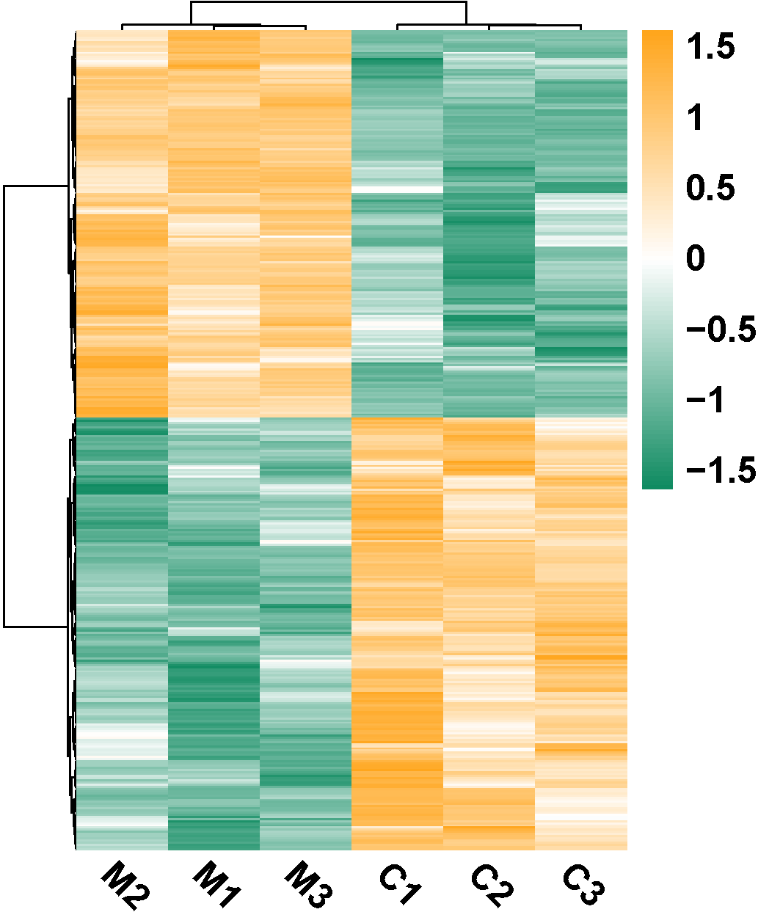


**Fig. S8** Heatmap of metabolomics differentially expressed metabolites


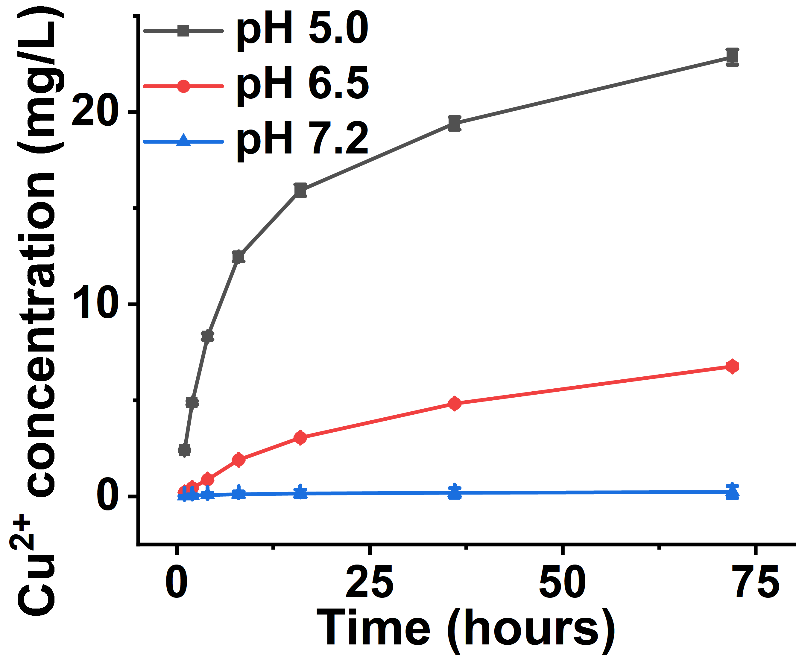


**Fig. S9** Cu SAs/MoS_2_ at different pH values in the ability to release Cu^2+^


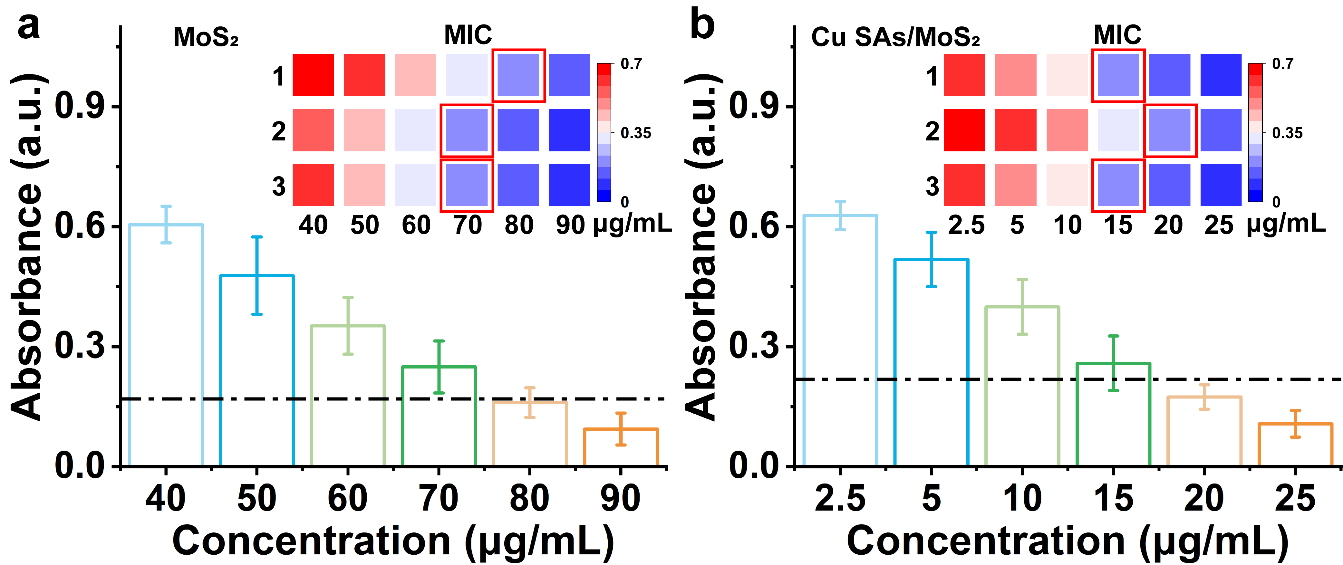


**Fig. S10** MIC of **a** MoS_2_ and **b** Cu SAs/MoS_2_ in *MRSA*


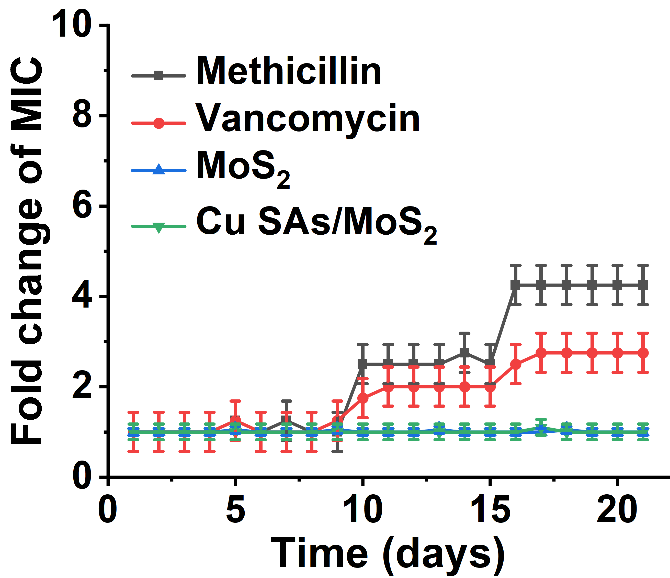


**Fig. S11** Evolution of indirect drug resistance of *MRSA* to MoS_2_, Cu SAs/MoS_2_, vancomycin, and methicillin after 21 days of cultivation in liquid medium


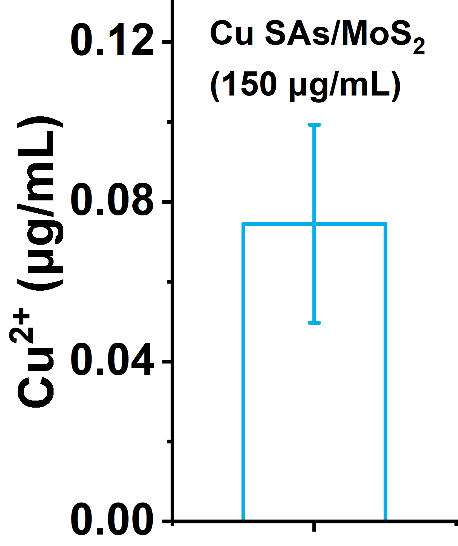


**Fig. S12** Detection of Cu^2+^ content in Cu SAs/MoS_2_ using ICP‒OES


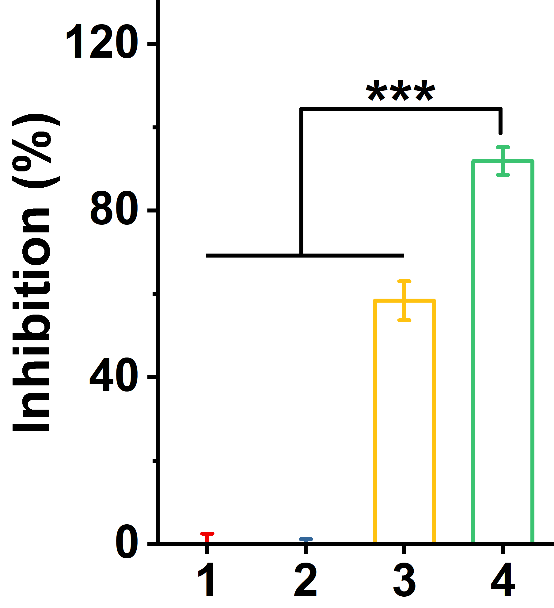


**Fig. S13** Rates of inhibition of biofilms in each group. 1: control; 2: H_2_O_2_; 3: MoS_2_ + H_2_O_2_; 4: Cu SAs/MoS_2_ + H_2_O_2_


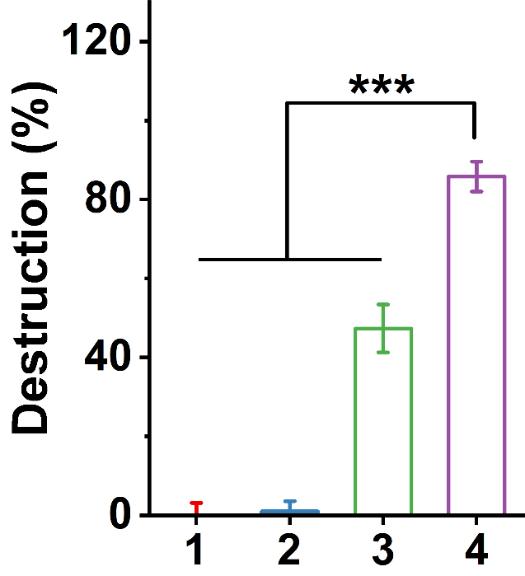


**Fig. S14** Rates of disruption of biofilms in each group. 1: control; 2: H_2_O_2_; 3: MoS_2_ + H_2_O_2_; 4: Cu SAs/MoS_2_ + H_2_O_2_


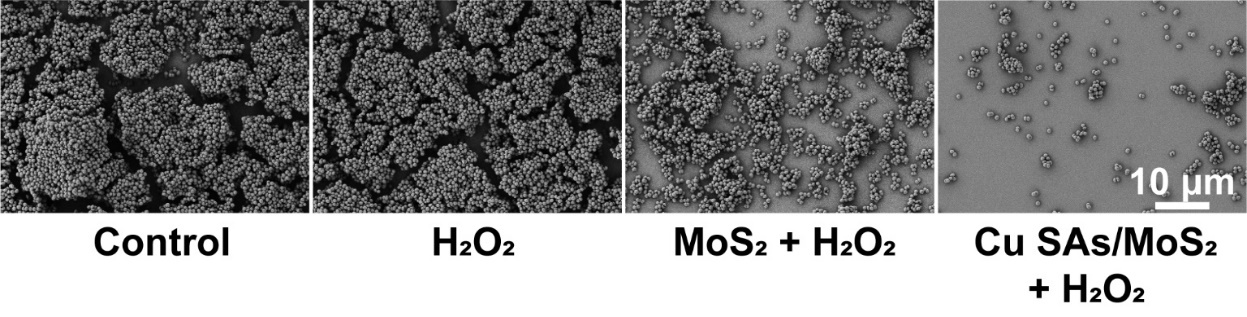


**Fig. S15** SEM images after biofilm treatment


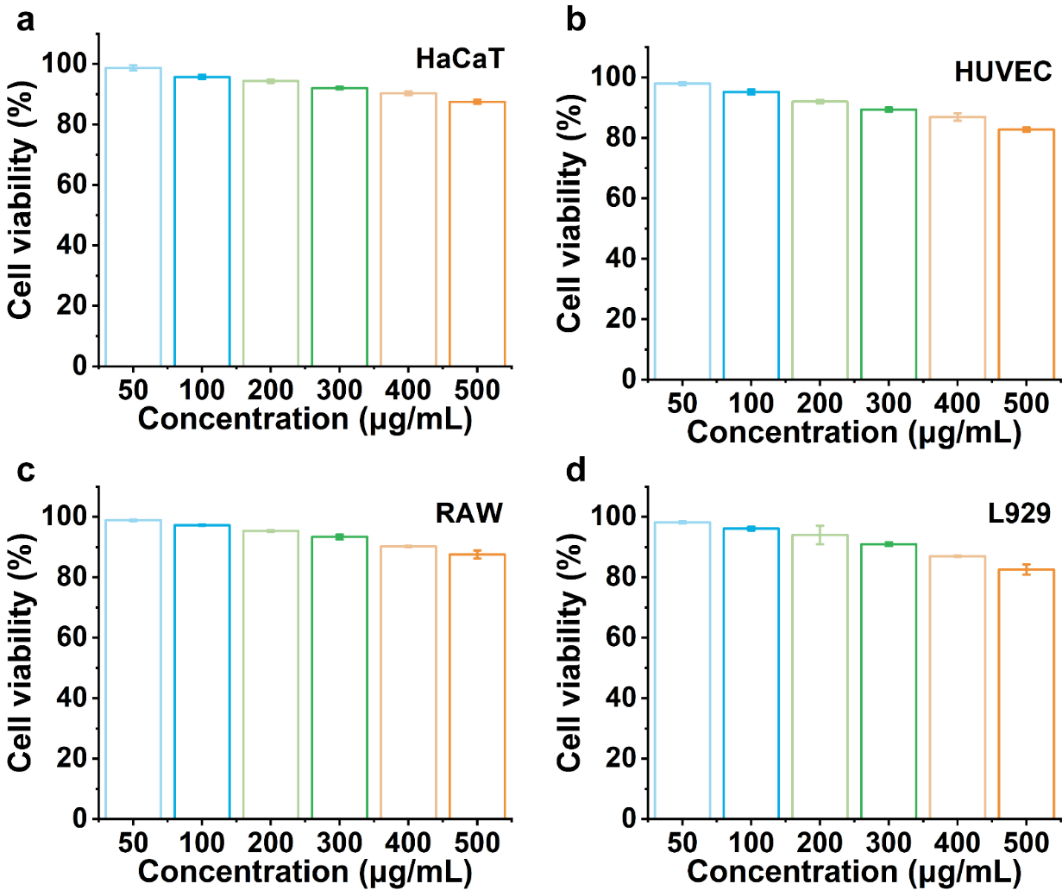


**Fig. S16** Cell viability of **a** HaCaT, **b** HUVCE, **c** RAW, and **d** L929 cells exposed to different concentrations of Cu SAs/MoS_2_


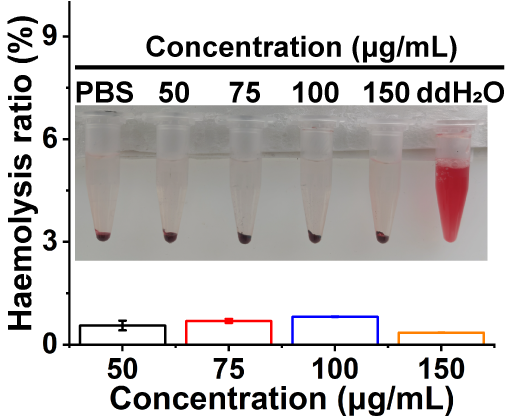


**Fig. S17** Hemolysis test at different concentrations of the Cu SAs/MoS_2_


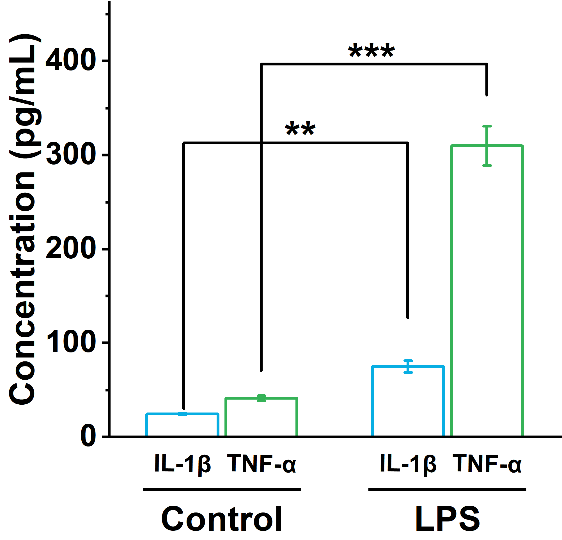


**Fig. S18** Assessment of the inflammatory microenvironment in macrophage culture


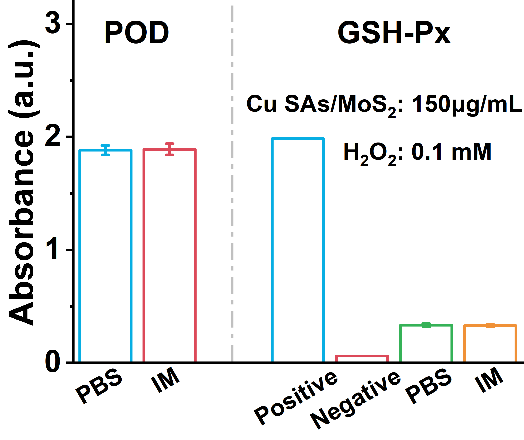


**Fig. S19** POD-like and GSH-Px-like activities in the IM


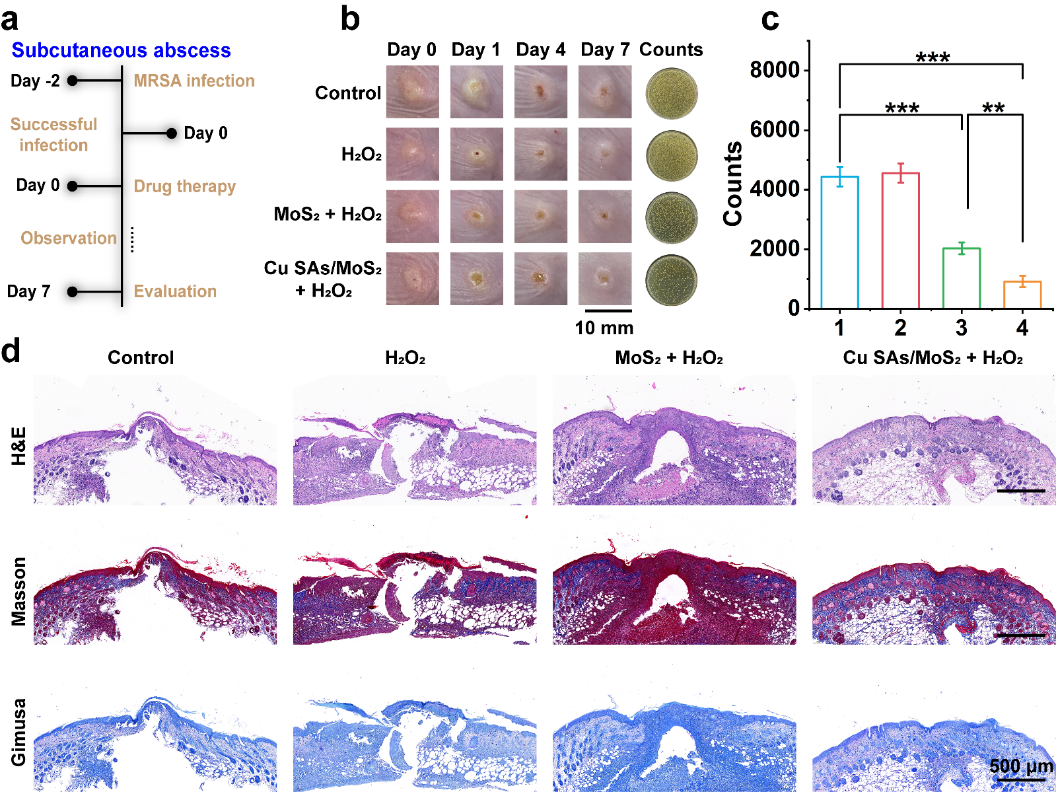


**Fig. S20 Cu SAs/MoS_2_ was evaluated for the treatment of subcutaneous abscesses. a** Schematic diagram of a subcutaneous abscess model of *MRSA* infection and its treatment. **b** Photos of the wound and plate photos of bacterial residues in the skin tissue on the seventh day. **c** Residual bacteria in wounds. **d** H&E staining, Masson staining, and Gemusa staining images of wounds after different treatments


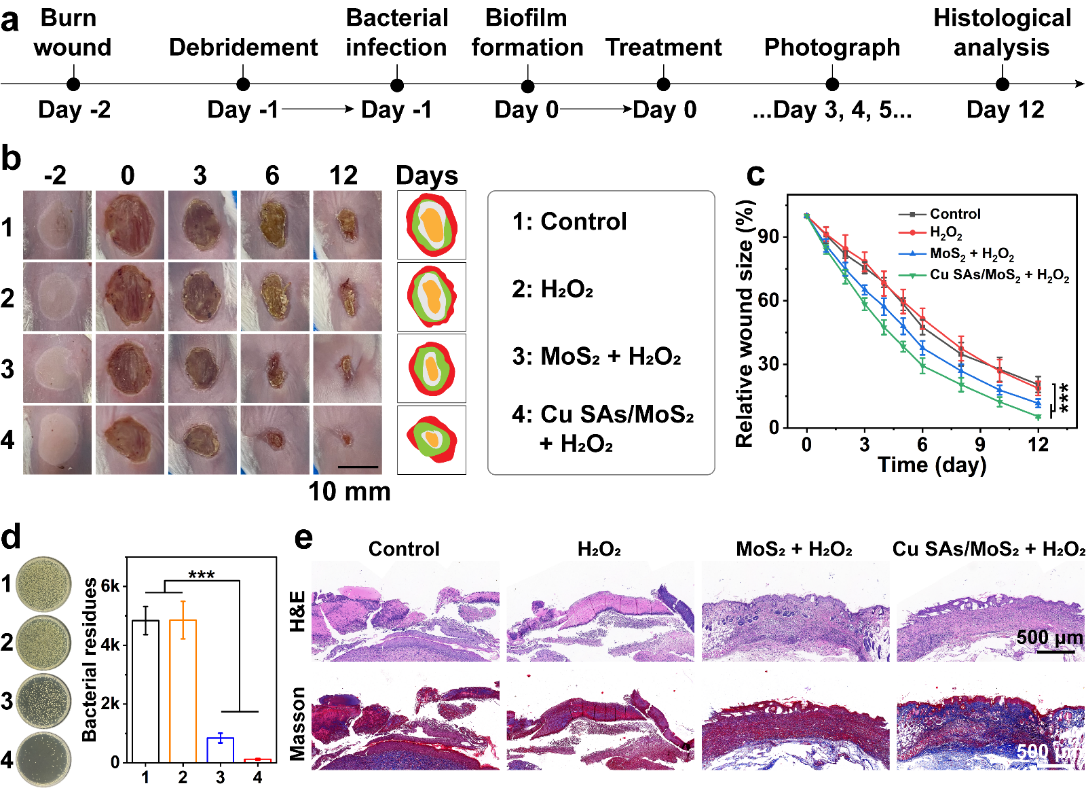


**Fig. S21 Cu SAs/MoS_2_ was evaluated for the treatment of burn wounds infected with *E. coli*. a** Schematic diagram of a burn wound model caused by *E. coli* infection and its treatment. **b** Twelve-day wound healing images and **c** wound healing area statistics. **d** Bacterial residues in skin tissue **e** H&E staining and Masson staining images of wounds after different treatments


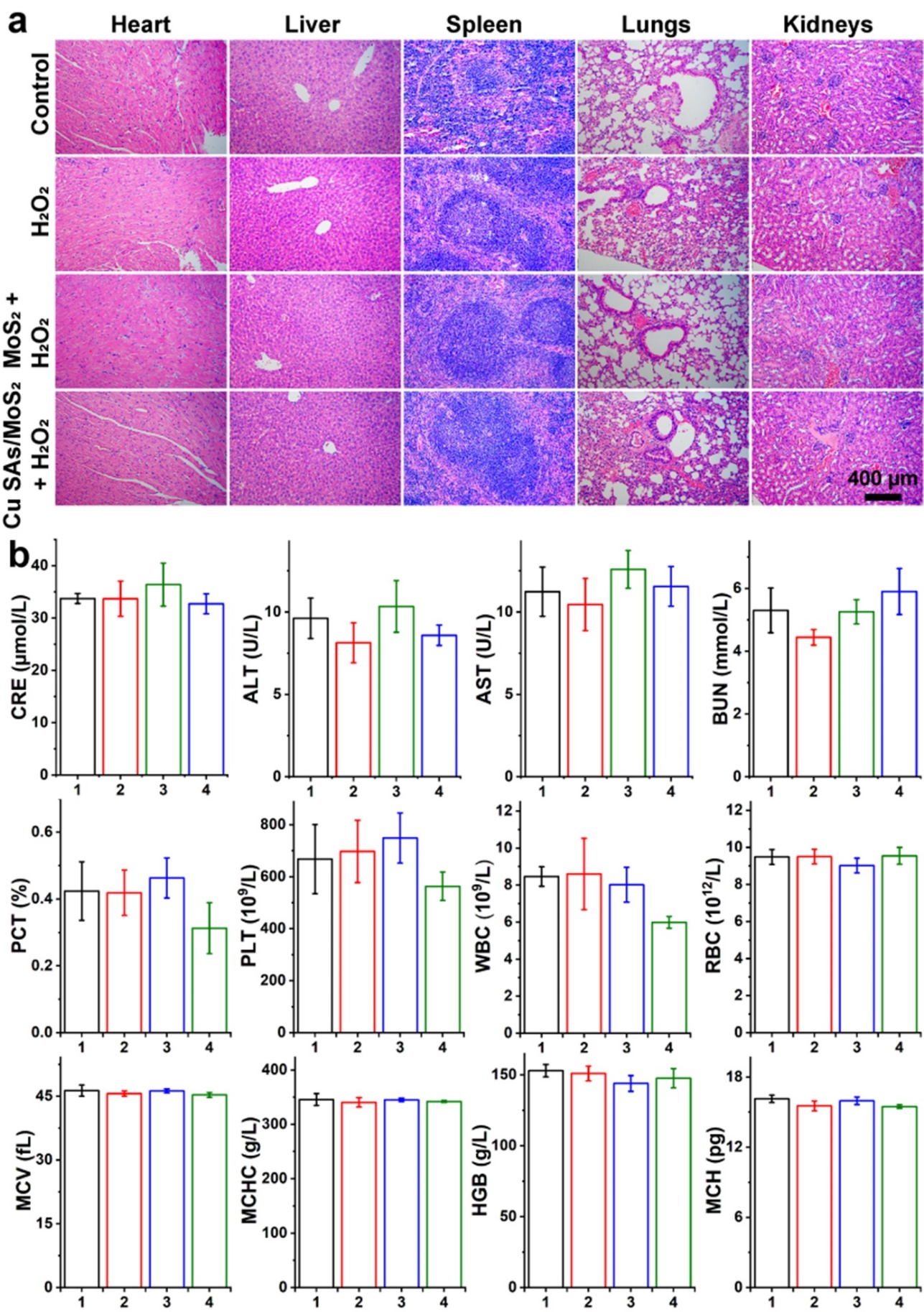


**Fig. S22 *In vivo* biocompatibility assessment of Cu SAs/MoS_2_*.* a** H&E sections of major organs (heart, liver, spleen, lung, and kidney) of mice in different groups on day 12. **b** Routine blood and biochemical parameters of normal mice and other treatment groups on day 12.


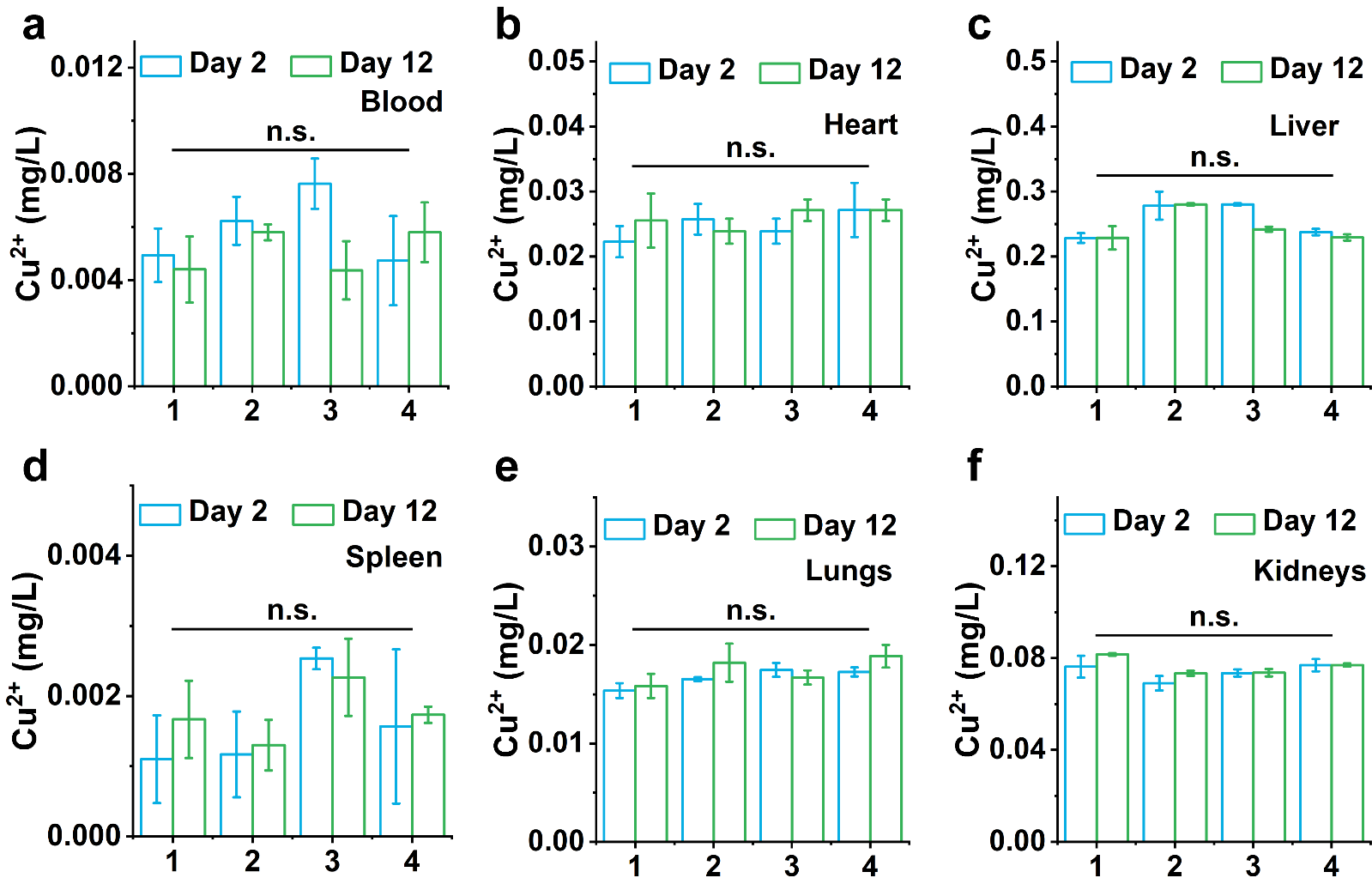


**Fig. S23** The concentrations of Cu^2+^ in the **a** blood and **b-c** organs (heart, liver, spleen, lungs and kidneys) were measured via ICP‒OES

**Table S1** Fitting data extracted from the Cu SAs/MoS_2_ FT-EXAFS

| Sample | Shell | *CN*^a^ | *R*(Å)^b^ | *σ*^2^(Å^2^)^c^ | Δ*E*^0^(eV)^d^ | *R* factor |
| --- | --- | --- | --- | --- | --- | --- |
| Cu SAs/MoS_2_ | Cu-S | 3.03 | 2.38 | 0.0109 | 3.8 | 0.0043 |

*CN*^a^, coordination number; ^b^*R*, distance between absorber and backscatter atoms; ^c^*σ*^2^, Debye‒Waller factor to account for both thermal and structural disorders; ^d^Δ*E*^0^, inner potential correction; *R* factor indicates the goodness of fit.
